# Influences of the heme-lysine crosslink in cytochrome P460 over redox catalysis and nitric oxide sensitivity†
†Electronic supplementary information (ESI) available: Experimental materials and methods, and supplementary figures and tables. See DOI: 10.1039/c7sc03450d


**DOI:** 10.1039/c7sc03450d

**Published:** 2017-11-07

**Authors:** Avery C. Vilbert, Jonathan D. Caranto, Kyle M. Lancaster

**Affiliations:** a Department of Chemistry and Chemical Biology , Baker Laboratory , Cornell University , Ithaca , NY 14853 , USA . Email: kml236@cornell.edu

## Abstract

Ammonia (NH_3_)-oxidizing bacteria (AOB) derive total energy for life from the multi-electron oxidation of NH_3_ to nitrite (NO_2_^–^). One obligate intermediate of this metabolism is hydroxylamine (NH_2_OH), which can be oxidized to the potent greenhouse agent nitrous oxide (N_2_O) by the AOB enzyme cytochrome (cyt) P460. We have now spectroscopically characterized a 6-coordinate (6c) {FeNO}^7^ intermediate on the NH_2_OH oxidation pathway of cyt P460. This species has two fates: it can either be oxidized to the {FeNO}^6^ that then undergoes attack by NH_2_OH to ultimately generate N_2_O, or it can lose its axial His ligand, thus generating a stable, off-pathway 5-coordinate (5c) {FeNO}^7^ species. We show that the wild type (WT) cyt P460 exhibits a slow nitric oxide (NO)-independent conversion (*k*_His-off_ = 2.90 × 10^–3^ s^–1^), whereas a cross-link-deficient Lys70Tyr cyt P460 mutant protein underwent His dissociation *via* both a NO-independent (*k*_His-off_ = 3.8 × 10^–4^ s^–1^) and a NO-dependent pathway [*k*_His-off(NO)_ = 790 M^–1^ s^–1^]. Eyring analyses of the NO-independent pathways for these two proteins revealed a significantly larger (*ca.* 27 cal mol^–1^ K^–1^) activation entropy (Δ*S*^‡^) in the cross-link-deficient mutant. Our results suggest that the Lys–heme cross-link confers rigidity to the positioning of the heme P460 cofactor to avoid the fast NO-dependent His dissociation pathway and subsequent formation of the off-pathway 5c {FeNO}^7^ species. The relevance of these findings to NO signaling proteins such as heme-nitric oxide/oxygen binding (H-NOX) is also discussed.

## Abstract 2

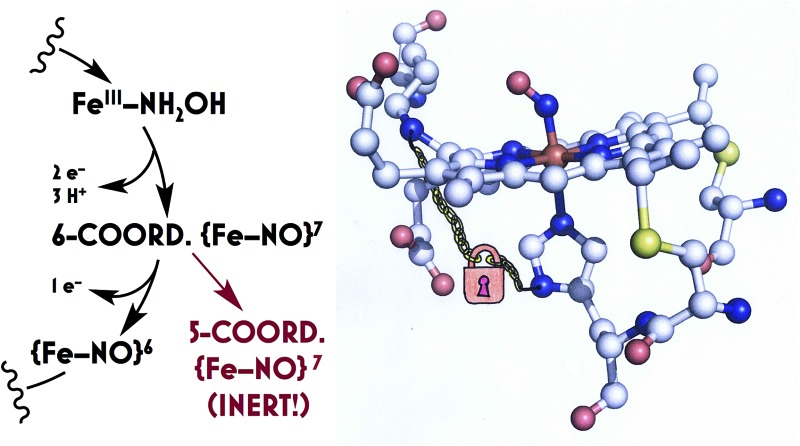
A vital role has been identified for the heme-lysine cross-link unique to cytochromes P460: preventing enzyme deactivation during catalysis by the obligate nitrification metabolite nitric oxide.

## Introduction

Ammonia (NH_3_)-oxidizing bacteria (AOB) derive energy for life from nitrification: the proton-coupled multi-electron oxidation of NH_3_ to nitrite (NO_2_^–^).^1^,^2^ Nitrification begins with the oxidation of NH_3_ to hydroxylamine (NH_2_OH) by the integral membrane enzyme ammonia monooxygenase. NH_2_OH is then oxidized to nitric oxide (NO) by the multi-heme enzyme hydroxylamine oxidoreductase (HAO) to establish net electron flow.^3^ The physiological means through which NO is oxidized to NO_2_^–^ are unknown. Detailed mechanistic understanding of controlled NH_2_OH oxidation is vital to the understanding of how nature uses NH_3_ as fuel.

**Fig. 1:**
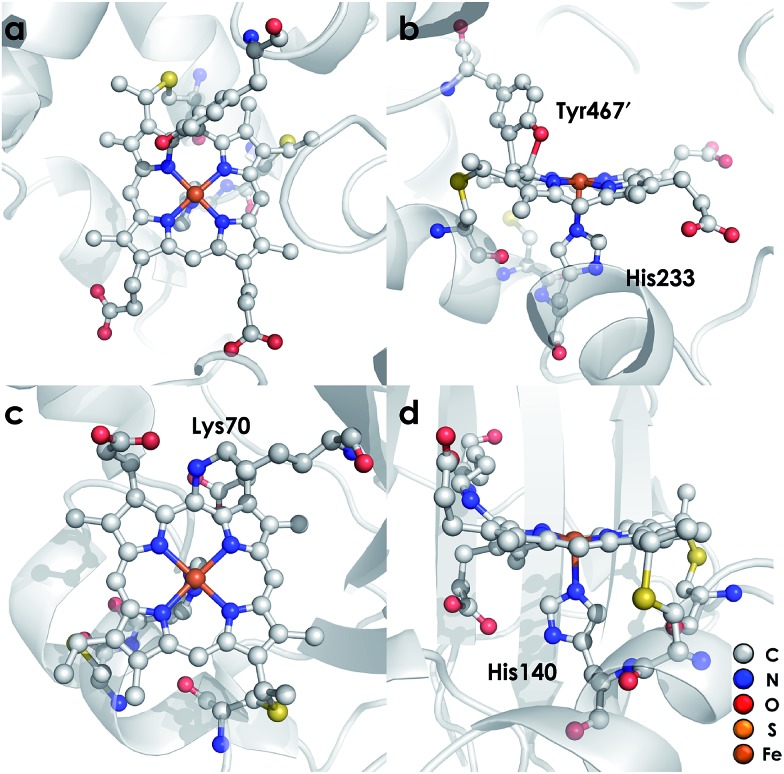
Views of the top (a) and side (b) of the *Nitrosomonas europaea* HAO heme P460 cofactor (2.1 Å resolution X-ray crystal structure, PDBID 4FAS) and the top (c) and side (d) of the *N. europaea* cytochrome (cyt) P460 heme P460 cofactor (1.8 Å resolution X-ray crystal structure, PDBID 2JE3). Both cofactors are c-heme cofactors with additional covalent amino acid side chain attachments. In HAO, Tyr467 from a neighboring subunit cross-links *via* the Cε at the 5′ meso carbon of the porphyrin and *via* the phenolate O at the neighboring pyrrole α-carbon. In cyt P460, the Lys70 amine N cross-links to the 13′ meso carbon.

HAO is a homotrimer of octaheme subunits.^4^ Seven of the hemes in each subunit are coordinatively saturated c-type hemes that mediate electron transfer. The remaining heme, also a c-type heme, is the site of NH_2_OH oxidation. This heme is called the heme P460 center because it has a characteristic Fe^II^ Soret absorption maximum at 463 nm.^5^ HAO heme P460 cofactors are unique in that they feature two cross-links with a tyrosine (Tyr) side chain (Fig. 1a and b). The Tyr Cε_1_ and phenolate O cross-link with the c-heme at the 5′ meso carbon and the adjacent pyrrole α-carbon, respectively.^4^ These attachments disrupt the π conjugation of the porphyrin ring and distort the planarity of the heme, resulting in a ruffled heme structure. P460 cofactors are found within enzymes from other bacteria as well, including methanotrophs^6^ and anaerobic NH_3_ oxidizers (anammox).^7^ The anammox bacterium *Kuenenia stuttgartiensis* contains at least 10 P460-containing HAO paralogs, at least two of which exhibit hydroxylamine or hydrazine oxidoreductase activity.^7^,^8^ Thus, the presence of P460 cofactors appears to be a hallmark of N-oxidation functionality.^9^ However, the characteristics that make this cofactor suitable for such reactions remain unknown.

**Fig. 2:**
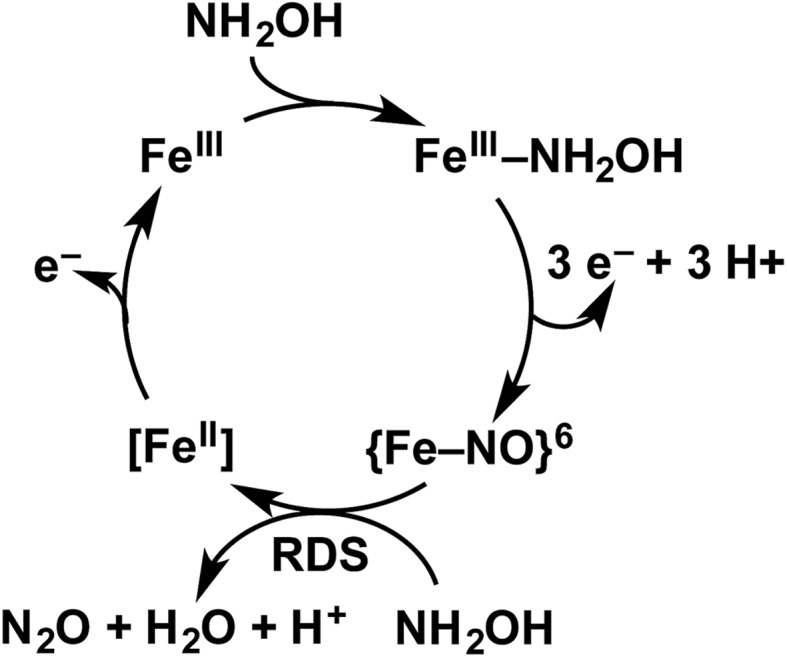
Working mechanism of the cyt P460 driven oxidation of NH_2_OH and formation of N_2_O. Adapted from ref. 13.

Detailed understanding of NH_2_OH oxidation by heme P460 centers is necessary to establish a link between the properties of this unique cofactor and its reactivity. However, spectroscopic probing of reaction intermediates at the 3 HAO heme P460 cofactors is occluded by the signals of the 21 electron transfer hemes. To overcome this challenge, we have explored the NH_2_OH oxidation mechanism of cytochrome (cyt) P460, a soluble, dimeric mono-heme enzyme found in the periplasms of AOB.^9^ In contrast to HAO, cyt P460 cofactors feature a Lys side chain amine covalently attached to the 13′ meso C of the c-heme (Fig. 1c and d).^10^ Despite this substitution, the UV-visible (UV-vis) absorption and ^57^Fe Mössbauer properties characteristic of Fe^II^ heme P460 are preserved.^11^ Cyt P460 had previously been reported to oxidize NH_2_OH to NO_2_^–^.^12^ However, we recently showed that cyt P460 oxidizes NH_2_OH selectively to nitrous oxide (N_2_O), not NO_2_^–^, under anaerobic conditions.^13^ In our reported working mechanism (Fig. 2), Fe^III^ cyt P460 binds NH_2_OH to form a stable Fe^III^–NH_2_OH adduct. This species then undergoes 3-electron oxidation to an {FeNO}^6^ intermediate (Enemark–Feltham^14^ notation denoting either Fe^III^–NO˙, Fe^II^–NO^+^, or Fe^IV^–NO^–^). Nucleophilic attack on this {FeNO}^6^ intermediate by a second equivalent of NH_2_OH produces N_2_O. Consequently, each NH_2_OH is oxidized by 2 electrons. Accounting for 2-electrons recycled to NH_3_ monooxygenase for turnover,^1^ such a cycle does not generate net cellular reducing equivalents for AOB.

The aforementioned reactivity prompted our reevaluation of the bacterial nitrification pathway. We noted that NO_2_^–^ was among the products of cyt P460 reactivity with NH_2_OH when the reactions were carried out aerobically.^13^ However, NO_2_^–^ concentrations were never stoichiometric with NH_2_OH input, likely owing to competition with the reaction producing N_2_O. We hypothesized that this NO_2_^–^ was produced as a result of the non-enzymatic reaction of aqueous NO with O_2_,^15^ implying that P460-driven NH_2_OH oxidation terminates at the {FeNO}^6^ intermediate. If NO loss could outcompete the reaction of this intermediate with NH_2_OH, the 3-electron oxidation of NH_2_OH would be assured and, thus, establish a net electron flow for AOB. Subsequent experiments confirmed that this outcome indeed occurs with HAO: NH_2_OH is enzymatically oxidized by 3 electrons to NO and then swiftly released.^3^

Thus, rapid access to the {FeNO}^6^ intermediate is essential for the N_2_O-generating mechanism of cyt P460 and the NO-generating mechanism of both cyt P460 and HAO. However, concerted biological 3-electron oxidation is unlikely. Moreover, the cyt P460 {FeNO}^6^ species is formed with either 1- or 2-electron oxidants.^13^ These observations strongly suggest that the oxidation of Fe^III^–NH_2_OH to the {FeNO}^6^ intermediate occurs *via* sequential 1- or 2-electron steps, or both. Proposed intermediates include a 1-electron-oxidized Fe^III^–NH_2_OH radical (Fe^III^–˙NH_2_OH) and a 2-electron-oxidized species formulated either as a ferric nitroxyl (Fe^III^–HNO) or its conjugate base {FeNO}^7^.^16^ However, no evidence for these intermediates has been provided. Electron paramagnetic resonance (EPR) spectroscopy provided evidence for a minor 5-coordinate (5c) {FeNO}^7^ species formed either when preparing Fe^III^–NH_2_OH samples or following complete oxidant consumption after multiple turnovers of cyt P460.^13^ This species was shown to be off-pathway. Hendrich and co-workers^17^ observed a similar off-pathway 5c {FeNO}^7^ species with EPR spectroscopy when fully reduced HAO was allowed to react with NH_2_OH.

Herein, we report the characterization of a 6-coordinate (6c) {FeNO}^7^ intermediate in *N. europaea* cyt P460 that is on-pathway and precedes the formation of the critical {FeNO}^6^ species. This 6c {FeNO}^7^ intermediate slowly decays in a NO-independent manner to the off-pathway 5c {FeNO}^7^ species. This conversion represents dissociation of the axial His140. Kinetic studies of a 13′ cross-link-deficient cyt P460 mutant (Lys70Tyr cyt P460) revealed that at least one function of this cross-link is to kinetically bypass the production of the off-pathway 5c {FeNO}^7^ intermediate during turnover by protecting the cofactor from deactivation by NO. The rate of 6c-to-5c conversion in the Lys70Tyr cyt P460 {FeNO}^7^ is accelerated by several orders of magnitude compared with the wild-type (WT) protein due to the mechanistic participation of excess NO. This rapid, NO-dependent 6c-to-5c {FeNO}^7^ conversion is reminiscent of the activation mechanism for heme-NO/O_2_ (H-NOX) binding proteins including soluble guanylate cyclase (sGC).^18^

## Results

### Identification of a cyt P460 6-coordinate {FeNO}^7^ species

**Fig. 3:**
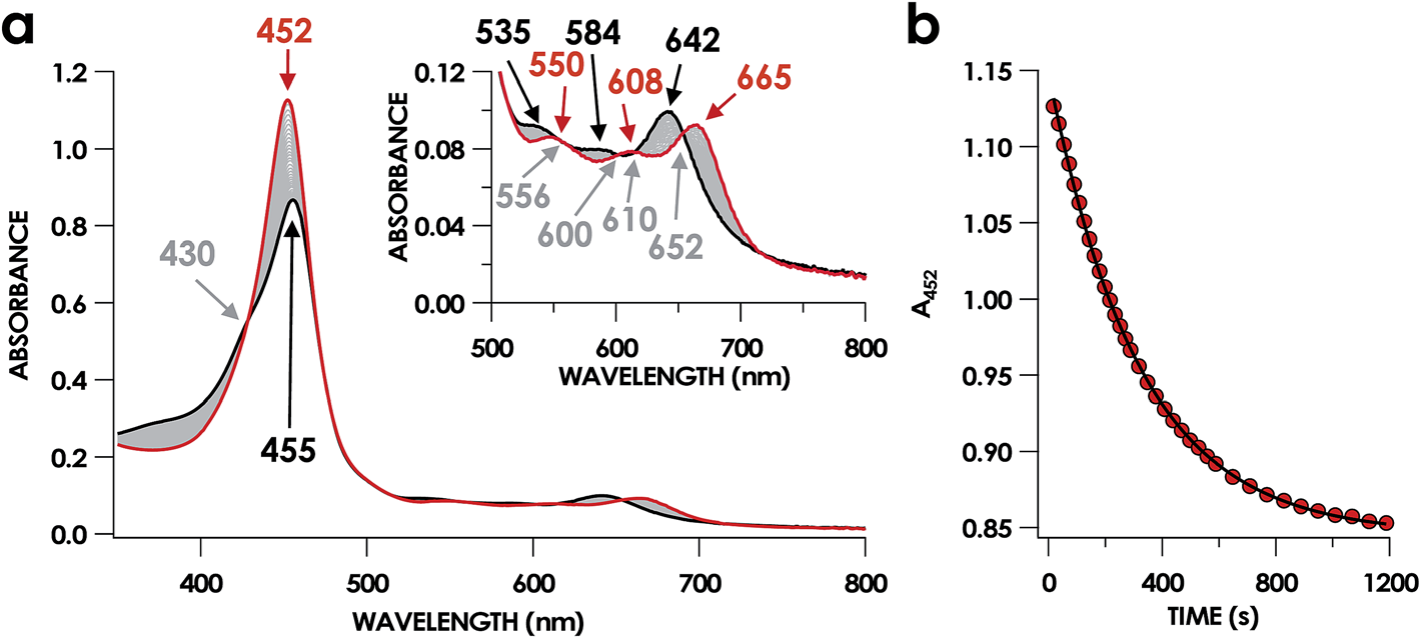
The 20 min UV-vis absorption full-spectral (a) and 452 nm single-wavelength (b) time courses of the reaction of 15 μM Fe^III^ cyt P460 and 600 μM HNO in 200 mM HEPES buffer (pH 8.0). In (a), the solid red trace is the spectrum collected immediately after mixing, the solid black trace is the spectrum collected after 20 min, and grey spectra were collected in 30 s increments. The inset highlights the time course in the Q-band region. Absorption maxima in nanometers are labeled with colors corresponding to each species. Isosbestic points are labeled in gray. In (b), the black trace is a single exponential (*A*_452_ = *y*_0_ + *A* × e^–*k*_obs_×*t*^) fit to the data, yielding *k*_obs_ = 3.15 × 10^–3^ s^–1^.

In our previous study,^13^ we generated an off-pathway 5c {FeNO}^7^ species. This species was generated *via* the treatment of Fe^III^ cyt P460 with the HNO donor disodium diazen-1-ium-1,2,2 triolate (Na_2_N_2_O_3_). One mole of Na_2_N_2_O_3_ liberates 1 mol of HNO with a half-life of 2 min when in room temperature pH 8.0 buffer. Hereafter, all HNO concentrations are expressed as the nominal final concentration expected from this Na_2_N_2_O_3_ decomposition. In the present work, monitoring of the UV-vis absorption spectral time course immediately after the treatment of 15 μM Fe^III^ cyt P460 with 100 μM HNO revealed a previously uncharacterized species. The new species forms within 2 min and exhibits a UV-vis absorption spectrum with a Soret maximum at 452 nm and Q-band maxima at 550, 608, and 665 nm (Fig. 3). This species decays within 15 min, resulting in a UV-vis absorption spectrum with a Soret maximum at 455 nm and Q-band maxima at 535, 584, and 642 nm. These absorption features correspond to the aforementioned 5c {FeNO}^7^ species.^13^ A similar spectral time course was observed when 15 μM Fe^II^ cyt P460 was treated with 100 μM NO generated by Proli-NONOate at pH 8.0. One mole of Proli-NONOate liberates 2 mol of NO with a half-life of 2 s in room temperature pH 8.0 buffer. Hereafter, all NO concentrations are expressed as the nominal final concentration expected from this Proli-NONOate decomposition. Isosbestic points observed in these spectral time courses at 430, 556, 600, 610, and 652 nm indicate a one-step conversion between the two species. Together, the data suggest that an uncharacterized species forms and slowly decays to the 5c {FeNO}^7^ species.

**Fig. 4:**
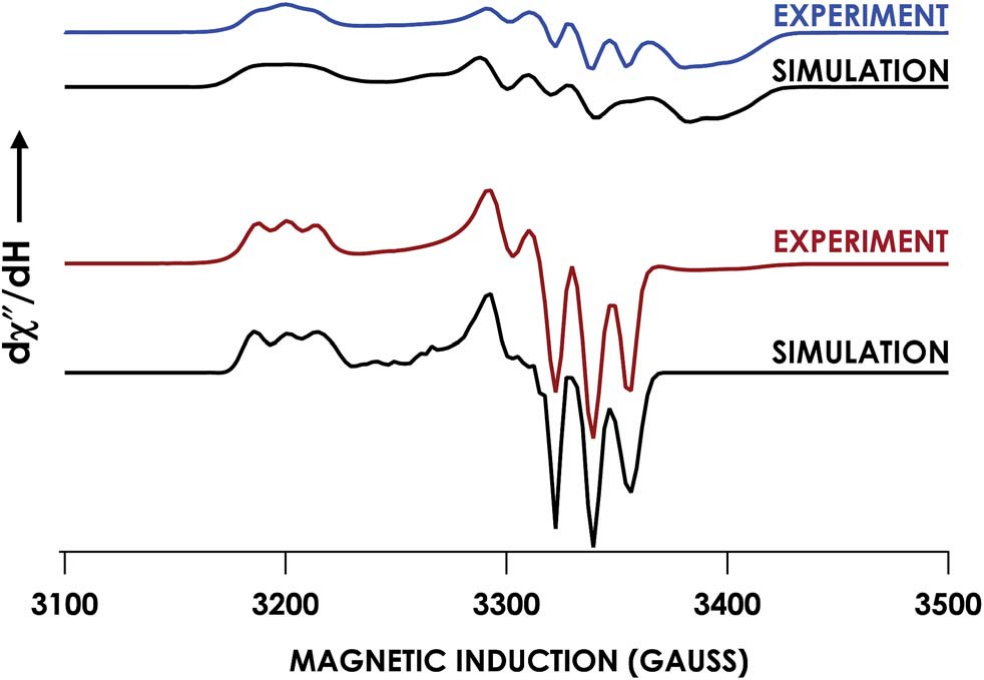
Continuous-wave X-band (9.40 GHz) EPR spectra of 200 μM Fe^III^ cyt P460 treated with 1 mM HNO in 200 mM HEPES buffer (pH 8.0) at room temperature and allowed to mature for 3 min (blue) or 30 min (red) before freezing. Measurements were obtained at 12 K with 63 μW microwave power. SpinCount simulations are shown in black for each spectrum. Spin Hamiltonian parameters for the blue spectrum, corresponding to the WT cyt P460 6c {FeNO}^7^ species, are (*g*_1_, *g*_2_, *g*_3_) = (2.10, 2.01, 1.98) and (^14^N *A*_1_, *A*_2_, *A*_3_) = (37, 55, 40 MHz). Parameters for the red spectrum, corresponding to the WT cyt P460 5c {FeNO}^7^ species are (*g*_1_, *g*_2_, *g*_3_) = (2.10, 2.03, 2.01) and (^14^N *A*_1_, *A*_2_, *A*_3_) = (50, 57, 45 MHz).

The spin state of this new species was characterized with continuous-wave X-band EPR (Fig. 4). Samples were prepared by treating 150 μM cyt P460 with 750 μM HNO in pH 8.0 buffer at 25 °C. The samples were frozen with liquid N_2_ within 3 min of mixing. The resulting EPR spectrum was consistent with an *S* = 1/2, 6c heme {FeNO}^7^ species: the simulated *g*-values were 2.10, 2.01, and 1.98 with corresponding ^14^N hyperfine values of 37, 55, and 40 MHz, respectively.^19^ The sample under the same reaction condition but frozen after 1 h had a distinct EPR spectrum with simulated *g*-values of 2.10, 2.03, and 2.01 and corresponding ^14^N hyperfine values of 50, 57, and 45 MHz, respectively. These parameters match those previously reported for the off-pathway 5c {FeNO}^7^ intermediate.^13^ Typically, 6c {FeNO}^7^ complexes exhibit a 9-line ^14^N superhyperfine splitting from the bound NO and the axially bound N(His). The lack of a 9-line ^14^N superhyperfine splitting from the bound Fe–N(His) could indicate either a weak Fe–N(His) bond or a large degree of disorder of the bound His.^20^ However, complementary spectroscopic data using techniques such as electron nuclear double resonance (ENDOR) would need to be acquired in order to accurately quantify the Fe–N(His) hyperfine interaction. These investigations are underway and will be reported elsewhere.

**Fig. 5:**
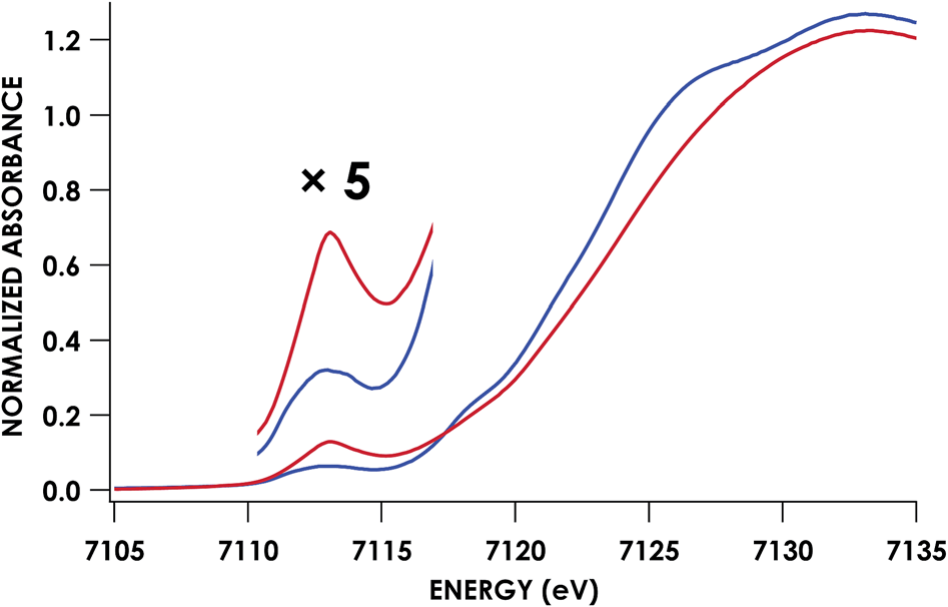
Fe K-edge XAS data obtained at 10 K for 1 mM cyt P460 6c (blue) and 5c (red) {FeNO}^7^ species in glassed 200 mM HEPES buffer (pH 8.0) containing 25% v/v glycerol. Note the 1s → 3d pre-edge feature at 7113.3 eV.

Fe K-edge X-ray absorption spectroscopy (XAS) data were obtained for both of these {FeNO}^7^ species. The Fe–K edge absorption near-edge regions of both the 6c and 5c {FeNO}^7^ species are shown in Fig. 5. The pre-edge feature near 7113 eV is conventionally assigned as a quadrupole-allowed Fe 1s → 3d transition that can gain intensity *via* an electric dipole mechanism.^21^ This feature appears at 7113.3 eV in the spectrum of the initially formed 6c {FeNO}^7^ intermediate. The feature exhibits significantly higher intensity in the 5c {FeNO}^7^ spectrum, and this intensity increase is consistent with decreased centrosymmetry at Fe: as the coordination number decreases, the attendant diminished centrosymmetry confers dipole allowedness to the 1s → 3d transition and a corresponding increase in pre-edge intensity.

Fits of the extended X-ray absorption fine structure (EXAFS) region from *k* values of 2–14 Å^–1^, where *k* is the photoelectron wave number, for the WT 6c and 5c {FeNO}^7^ species give short Fe–N scatters assignable to Fe–NO at 1.86 Å and 1.74 Å, respectively (Fig. S9† and Table 1). These distances are consistent with typical Fe–NO bond lengths in 6c and 5c heme {FeNO}^7^ species, respectively.^22^ The data resolution precluded the fitting of an Fe–N(His) scattering path in the 6c {FeNO}^7^ species independent from the Fe–N(heme) paths. However, the EXAFS were best fit for this species with 5 rather than 4 Fe–N scatters at 2.04 Å. Moreover, we could reliably fit the axial His of the 5c {FeNO}^7^ intermediate at a distance 2.53 Å, which is well outside the range of coordination.

**Table 1:** Best fits to Fe K-edge EXAFS data obtained for WT cyt P460 5c and 6c {FeNO}^7^ and Lys70Tyr 5c {FeNO^7^}^*a*^

	Scattering path	Coordination number	*R* (Å)	Δ*R* (Å)	*σ* ^2^	Δ*σ*^2^	*F* (%)
WT 6c {FeNO}^7^	Fe–N(pyrrole)	5	2.037	0.002	0.00504	0.00023	29.5
	Fe–N(NO)	1	1.858	0.012	0.00739	0.00153	
WT 5c {FeNO}^7^	Fe–N(pyrrole)	4	2.021	0.002	0.00271	0.000152	30.3
	Fe–N(NO)	0.75	1.735	0.023	0.01294	0.00363	
	Fe–N(His)	1	2.525	0.012	0.00381	0.00131	
Lys70Tyr 5c {FeNO}^7^	Fe–N(pyrrole)	4	1.991	0.003	0.00275	0.00022	35.7
	Fe–N(NO)	1	1.805	0.014	0.00693	0.00177	
	Fe–N(His)	1	2.483	0.012	0.00259	0.00122	

^*a*^EXAFS data were fit in OPT using paths calculated by FEFF7. Coordination numbers were held constant, whereas distances (*R*) and Debye–Waller factors (*σ*^2^) were allowed to float. Errors in coordination numbers are estimated to be on the order of 25%. Fits were performed over the entire Fourier transform window (0–6.0 Å). Goodness of fit was measured with F, which was defined as 
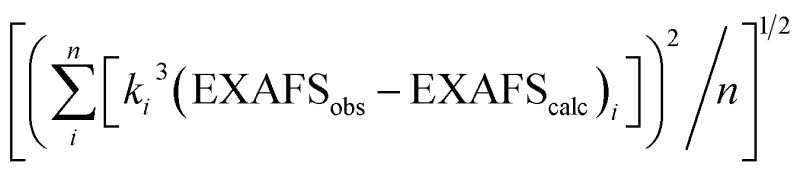
.

The combined UV-vis absorption, EPR, and X-ray absorption data reveal that Fe^III^ cyt P460 reacts with HNO to accumulate a 6c {FeNO}^7^ species, which subsequently decays to a 5c {FeNO}^7^ form within 30 min. The simplest interpretation of this data is that the conversion results from the dissociation of the axial His140. Dissociation of the ligand trans to the NO is frequently observed for heme {FeNO}^7^ species and is attributed to the trans influence of NO.^23^–^27^

### Cyt P460 6c {FeNO}^7^, a NH_2_OH oxidation intermediate

The characterized 6c-to-5c {FeNO}^7^ conversion suggests that the 5c {FeNO}^7^ species observed in our previous work arises from the slow decay of the 6c {FeNO}^7^ intermediate generated during cyt P460 turnover. Our previous failure to observe the 6c {FeNO}^7^ suggests that this intermediate reacts with either an oxidant or NH_2_OH, both present under turnover conditions, to form the {FeNO}^6^ intermediate.

To test whether the 6c {FeNO}^7^ species can be oxidized to the {FeNO}^6^ species, the former was allowed to react with an oxidant or NH_2_OH. To generate the 6c {FeNO}^7^ species, we treated 15 μM Fe^III^ cyt P460 with 200 μM HNO in anaerobic pH 8.0 buffer at room temperature for 2 min to produce 15 μM of cyt P460 6c {FeNO}^7^. The addition of 3 mM NH_2_OH to this solution resulted in no changes to the UV-vis absorption spectrum or the rate of 6c-to-5c {FeNO}^7^ conversion (Fig. S1†). These results suggest that NH_2_OH is unreactive with the 6c {FeNO}^7^ species.

**Fig. 6:**
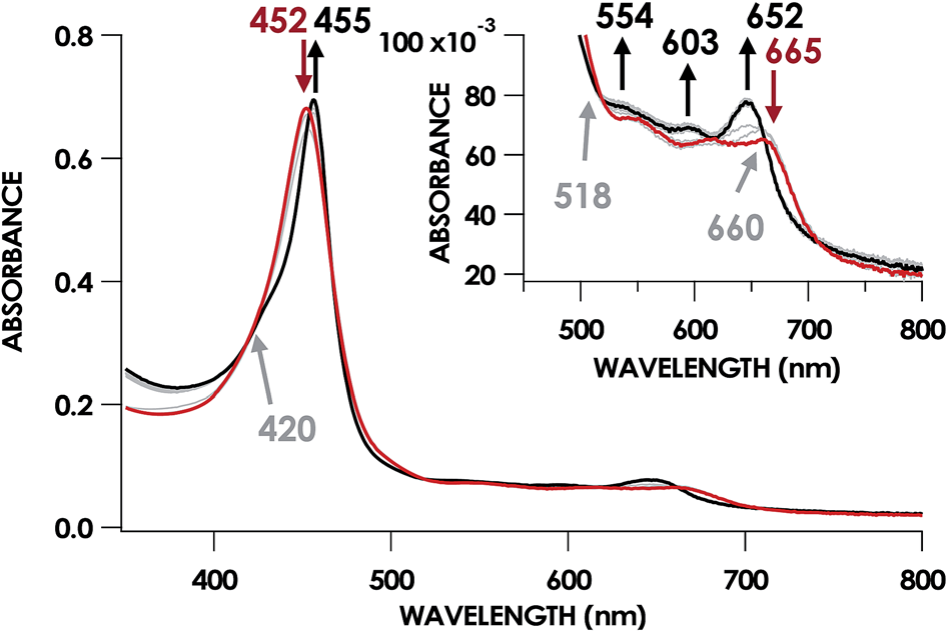
UV-vis absorption spectral time course of 15 μM 6c cyt P460 {FeNO}^7^ treated with Ru(NH_3_)_6_Cl_3_ in anaerobic 50 mM HEPES buffer (pH 8.0) at room temperature. The solid red trace is the absorbance spectrum collected 5 min after adding 10 equiv. of the HNO donor Na_2_N_2_O_3_ to 15 μM Fe^III^ cyt P460. The solid black trace shows the final spectrum collected after the addition of 15 equiv. of Ru(NH_3_)_6_Cl_3_ to these solutions and matches the previously reported spectrum of the cyt P460 {FeNO}^6^ intermediate. Grey spectra were collected in 30 s increments. The inset highlights the time course in the Q band region.

By contrast, oxidant addition promoted rapid changes in the UV-vis absorption spectrum. Anaerobic treatment of 15 μM cyt P460 6c {FeNO}^7^ with 100 μM of PMS [phenazinemethosulfate, *E*^0^ = +92 mV *vs.* normal hydrogen electrode (NHE)], DCPIP (2,6-dichlorophenolindophenol, *E*^0^ = +224 mV *vs.* NHE), or [Ru(NH_3_)_6_]Cl_3_ (*E*^0^ = +51 mV *vs.* NHE) effected 6c {FeNO}^7^ decay within 30 s (Fig. 6). The product of this reaction had UV-vis absorption features identical to those assigned to the cyt P460 {FeNO}^6^ species.^13^ Previous experiments showed that treating cyt P460 5c {FeNO}^7^ with these oxidants afforded no evidence of {FeNO}^6^ formation.^13^ The only spectral changes observed were a minor decrease in the 455 nm Soret maximum and the appearance of a shoulder at 414 nm, which suggested cofactor degradation (Fig. S2†). No conversion to {FeNO}^6^ was observed even after treatment with the far more potent oxidant potassium hexachloroiridate K_2_[IrCl_6_] (*E*^0^ = +892 mV *vs.* NHE). Here again, only degradation occurred.

EPR spectra obtained for cyt P460 6c and 5c {FeNO}^7^ samples treated with oxidant (Fig. S3a and b†) corroborated the results of the UV-vis absorption experiments. Under anaerobic conditions, 600 μM PMS was added to a solution of 200 μM 6c cyt P460 {FeNO}^7^ at pH 8.0 and 25 °C, and the mixture was immediately frozen in liquid N_2_. The resulting EPR spectrum was dominated by a sharp signal at *g* = 2.0 attributed to PMS semiquinone. The spectrum lacks features assigned to the 6c {FeNO}^7^ species and shows no evidence for any other Fe-based signals. This outcome suggests that most of the cyt P460 Fe is in an EPR-silent state, which is consistent with the oxidation of the 6c {FeNO}^7^ species to the EPR-silent {FeNO}^6^ form. Moreover, the EPR spectrum obtained after the addition of 600 μM PMS to 200 μM 5c cyt P460 {FeNO}^7^ at pH 8.0 and 25 °C shows EPR features consistent with those of the 5c {FeNO}^7^ species as well as the *g* = 2.0 signal assigned to PMS semiquinone. This PMS semiquinone spectrum is observed in samples of PMS in buffer, indicating that the semiquinone form is present even in the absence of protein or NH_2_OH. The aggregate data are consistent with the 5c {FeNO}^7^ species being unreactive and off-pathway; however, the 6c {FeNO}^7^ species can be oxidized to {FeNO}^6^. The 5c species therefore must have a more positive reduction potential than the 6c {FeNO}^7^. We rationalize that this is largely attributable to electron donation from His producing a more electron-rich FeNO unit. These data suggest that 6c {FeNO}^7^ is an intermediate of NH_2_OH oxidation by cyt P460.

To demonstrate conclusively that cyt P460 6c {FeNO}^7^ is a NH_2_OH oxidation intermediate, we generated this species by oxidizing the Fe^III^–NH_2_OH species. The 6c-to-5c {FeNO}^7^ conversion occurs on a minutes timescale, whereas oxidant rapidly converts this species to {FeNO}^6^ on a seconds timescale. Therefore, if 6c {FeNO}^7^ is generated as a catalytic intermediate, the presence of oxidant will kinetically favor its conversion to {FeNO}^6^ over His dissociation to form the off-pathway 5c {FeNO}^7^ species. Oxidation to {FeNO}^6^ should be first-order with respect to oxidant concentration. Therefore, at low oxidant concentrations, the rate of the oxidation pathway will be slow enough for the His dissociation pathway to kinetically compete. By these rationales, the 6c {FeNO}^7^ intermediate should accumulate immediately after the depletion of the oxidant. To test this hypothesis, we allowed 200 μM cyt P460 to react with 1 mM DCPIP and 1 mM NH_2_OH at pH 8.0 and room temperature. The sample was frozen in liquid N_2_ within 2 min, a time immediately after the blue color of the DCPIP disappeared. The 20 K EPR spectrum of this sample exhibited one anisotropic *S* = 1/2 signal with features identical to those observed for 6c {FeNO}^7^ (Fig. S4†). The signal accounts for 40 μM or 20% of the Fe centers in the sample. In the absence of any other Fe-based signal, we accounted for the remainder of the iron (160 μM) as EPR-silent {FeNO}^6^. Our inability to detect the 6c {FeNO}^7^ species within UV-vis absorption time courses likely results from the low ratio of 6c {FeNO}^7^ to {FeNO}^6^ concentrations and the overlapping UV-vis absorption features of the two species. Nevertheless, the EPR spectra clearly show evidence for the formation of the 6c {FeNO}^7^ species, and therefore, we assigned 6c {FeNO}^7^ as an intermediate on the cyt P460 NH_2_OH oxidation pathway.

### NO-independent His140 dissociation from cyt P460 {FeNO}^7^

NO promotes rapid (milliseconds to seconds) His dissociation in many heme proteins.^18^,^20^ This phenomenon underlies the mechanism of signal transduction by H-NOX proteins and sGC in both eukaryotes and bacteria.^28^ Given this common behavior by heme proteins, we sought to test if the rate of His dissociation from the cyt P460 6c {FeNO}^7^ is also promoted by NO.

**Fig. 7:**
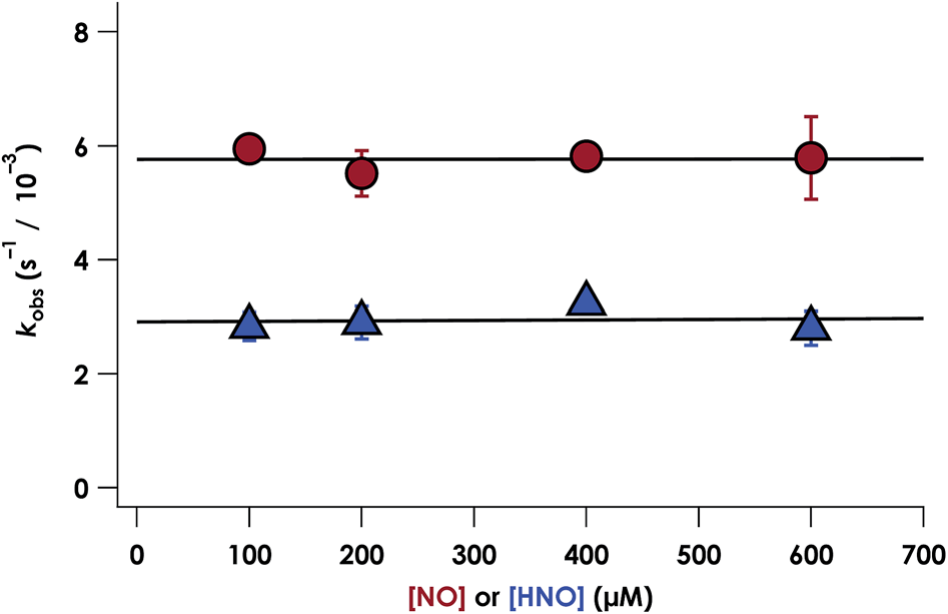
Plot of *k*_obs_*vs.* NO concentration (red circles) or HNO concentration (blue triangles). The corresponding *k*_His-off_ values are 2.9 ± 0.2 × 10^–3^ s^–1^ and 5.7 ± 0.2 × 10^–3^ s^–1^ for HNO and NO, respectively.

The cyt P460 6c {FeNO}^7^ species can be generated from the reaction of either Fe^III^ cyt P460 with HNO or Fe^II^ cyt P460 with NO (Fig. S5† and 7). The two independent methods allows for testing the reactivity of 6c {FeNO}^7^ in the absence or presence of a large excess of NO, respectively. Rate constants for 6c-to-5c {FeNO}^7^ conversion were obtained from the reaction of 15 μM Fe^II^ cyt P460 with varying excess concentrations of NO ranging from 100–600 μM. The conversion was monitored by the decrease in absorbance of the 6c {FeNO}^7^ Soret band at 452 nm. Single-exponential functions were fit to the 452 nm traces by nonlinear least-squares regression to determine *k*_obs_ under each condition. Plotting *k*_obs_ values *versus* NO concentration revealed that the 6c-to-5c {FeNO}^7^ conversion is zeroth-order in NO with a His140 dissociation rate constant (*k*_His-off_) of 5.7 ± 0.2 × 10^–3^ s^–1^. A similar *k*_His-off_ of 2.9 ± 0.2 × 10^–3^ ± s^–1^ was obtained when Fe^III^ cyt P460 was treated with HNO (Fig. 7). The data clearly show that His140 dissociation for cyt P460 6c {FeNO}^7^ proceeds *via* a mechanism that is independent of either NO or HNO. This behavior contrasts starkly with the behavior common to NO-sensing heme proteins. Furthermore, the His dissociation of cyt P460 is appreciably slower than that observed for other heme proteins, which dissociate their axial His on millisecond time scales. This slow His dissociation allows the oxidation of 6c {FeNO}^7^ to {FeNO}^6^ to kinetically outcompete the formation of the off-pathway 5c {FeNO}^7^ intermediate, and thus, appears essential to preserving active catalyst.

### Characterization of {FeNO}^7^ species on a cross-link deficient mutant, Lys70Tyr cyt P460

**Fig. 8:**
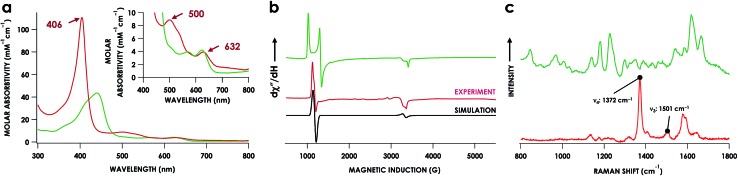
UV-vis absorption (a) EPR (b), and rR (c) spectra of Fe^III^ WT (green) and Lys70Tyr (red) cyt P460. Inset in (a) highlights the Q-band region. EPR *g*-values for Lys70Tyr cyt P460 are 5.78 and 1.98; E/D = 0.00. EPR spectra were measured at 9.40 GHz and 12 K with 633 μW microwave power. The rR data were obtained *via* near-resonance excitation with Soret absorption bands: *λ*_ex_ = 458.7 nm (20 mW) and 405.0 nm (20 mW) for the WT and Lys70Tyr cyt P460, respectively.

The lack of an NO-dependent *k*_His-off_ for cyt P460 6c {FeNO}^7^ prompted us to investigate how this anomalous behavior relates to the unique P460 cofactor structure. To this end, we hypothesized that the distinguishing Lys70 cross-link to the 13′ meso C of the P460 cofactor may influence *k*_His-off_. Therefore, we generated a cross-link-deficient Lys70Tyr cyt P460 mutant for a comparison of His140 dissociation kinetics. Purified Fe^III^ Lys70Tyr cyt P460 is a red protein with a UV-vis absorption Soret maximum at 406 nm and Q-bands at 500 nm and 632 nm (Fig. 8a). The continuous-wave X-band EPR spectra of the resting Fe^III^ exhibited an *S* = 5/2 signal with *g*-values of 5.78 and 1.98 (Fig. 8b). These *g*-values are consistent with an axial (E/D = 0.00) signal and suggest an increased heme symmetry compared with the WT Fe^III^ cyt P460 *S* = 5/2 spectrum with an E/D of 0.03. This increased symmetry is consistent with the loss of the Lys cross-link in the mutant.

To characterize the cofactor in the Lys70Tyr variant further, we obtained resonance Raman (rR) spectra *via* excitation near the Soret maxima of Fe^III^ WT (*λ*_ex_ = 457.8 nm) and Fe^III^ Lys70Tyr (*λ*_ex_ = 405.0 nm) cyt P460 (Fig. 8c). Detailed analysis of the Fe^III^ WT cyt P460 rR spectrum was beyond the scope of the present work; however, the increased number of observed bands relative to non-cross-linked hemes suggests that the cyt P460 cofactor has diminished symmetry. This increase in band count is consistent with the rR spectrum obtained for the HAO Fe^II^ heme P460 cofactor.^29^ The rR spectrum obtained for Fe^III^ Lys70Tyr cyt P460 exhibited an oxidation state marker band (*ν*_4_) at 1370 cm^–1^ but a spin-state marker band (*ν*_3_) at 1501 cm^–1^. Typically, *ν*_3_ greater than 1500 cm^–1^ indicates a low spin 6-coordinate heme, however, 5-coordinate ferric cyt c′ proteins—whose coordination was verified by EPR spectroscopy and crystal structures—also exhibit *ν*_3_*ca.* 1500 cm^–1^.^30^ The EPR spectrum of the Fe^III^ Lys70Tyr cyt P460 is also consistent with a high-spin ferric heme. To further characterize the coordination number of the mutant cyt P460, we also obtained the rR spectrum obtained of Fe^II^ Lys70Tyr cyt P460 (*λ*_ex_ = 405.0 nm), which has the profile of a standard, effectively *D*_4h_ 5c high spin Fe^II^ c-heme with *ν*_4_ at 1356 cm^–1^ and *ν*_3_ at 1473 cm^–1^ (Fig. S13†).^30^–^32^ The aggregate spectroscopic data are consistent with the restoration of a canonical c-heme due to loss of the 13′ cross-link.^33^

The Fe^III^ Lys70Tyr cyt P460 binds NH_2_OH to form the Fe^III^–NH_2_OH adduct (Fig. S14†). However, this mutant protein is incapable of turnover, resulting from loss of the Fe^III^–NH_2_OH oxidation reactivity. Therefore, the cross-link is necessary for reactivity of the Fe^III^–NH_2_OH adduct. This lack of NH_2_OH oxidation reactivity in the mutant will be addressed elsewhere.

**Fig. 9:**
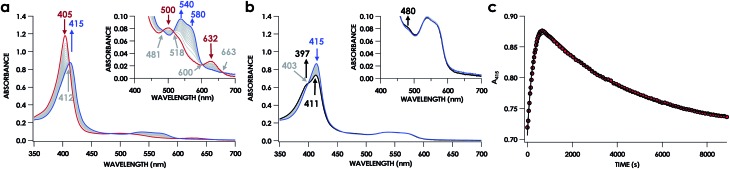
The 150 min UV-vis absorption full-spectral (a and b) and 415 nm single-wavelength time courses (c) of the reaction of 10 μM Fe^III^ cyt P460 with 100 μM of HNO in 200 mM HEPES buffer (pH 8.0). In (a) and (b), the solid red trace is the spectrum collected immediately after mixing, the solid blue trace is collected at 10 min and the solid black trace was collected at 150 min. Grey spectra were collected in 1 min increments. The insets highlight the time courses in the Q-band region. Absorption maxima in nanometers are labeled with colors corresponding to each species. Isosbestic points are labeled in gray. In (c), the black trace is a double-exponential fit (*A*_415_ = *A*_0_ + *A*_1_ × e^–k_obs(1)_×*t*^ + *A*_2_ × e^–k_obs(2)_×*t*^) to the data, yielding *k*_obs(1)_ = 3.7 × 10^–3^ s^–1^ and *k*_obs(2)_ = 2.7 × 10^–4^ s^–1^.

UV-vis absorption time courses of 6c {FeNO}^7^ formation and decay were obtained to compare the His dissociation between WT and Lys70Tyr cyt P460. Anaerobic treatment of 10 μM Fe^III^ Lys70Tyr cyt P460 with 600 μM HNO resulted in the appearance of a new species with an UV-vis absorption Soret maximum at 415 nm and Q-band maxima at 540 and 580 nm (Fig. 9). This species decayed slowly (within 80 min) to a species exhibiting a Soret peak at 413 nm with a shoulder at 396 nm and unshifted Q-bands at 540 and 580 nm. By contrast, no intermediate was observed when 10 μM Fe^II^ Lys70Tyr cyt P460 was treated with 600 μM NO. The UV-vis spectral time course showed the immediate formation of a stable species within the time of manual mixing. The absorption spectrum of this product matches that of the HNO reaction product, which suggests an identical Fe product for both reactions.

**Fig. 10:**
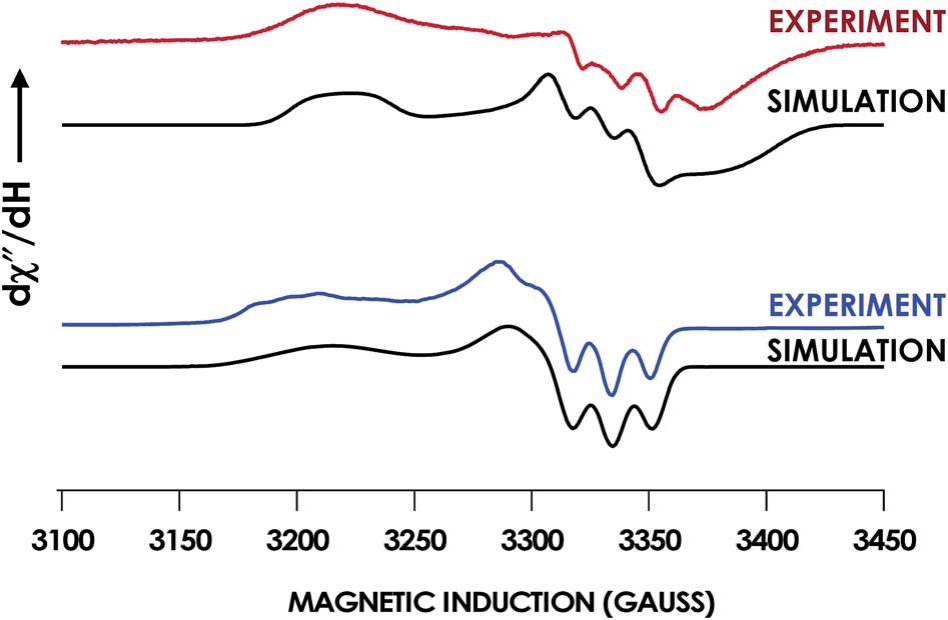
Continuous-wave X-band (9.40 GHz) EPR spectra measured at 8 K with 63 μW microwave power of 150 μM Fe^III^ Lys70Tyr cyt P460 treated with 750 μM HNO (red) or 150 μM Lys70Tyr Fe^II^ cyt P460 treated with 750 μM NO in 200 mM HEPES buffer pH 8.0 (blue). SpinCount simulations are shown in black for each spectrum. Spin Hamiltonian parameters for the red spectrum, corresponding to Lys70Tyr cyt P460 6c {FeNO}^7^, are (*g*_1_, *g*_2_, *g*_3_) = (2.09, 2.02, 1.98) and (^14^N *A*_1_, *A*_2_, *A*_3_) = (45, 47, 40 MHz). Parameters for the blue spectrum, corresponding to WT cyt P460 5c {FeNO}^7^, are (*g*_1_, *g*_2_, *g*_3_) = (2.09, 2.03, 2.01) and (^14^N *A*_1_, *A*_2_, *A*_3_) = (47, 41, 49 MHz).

EPR spectroscopic analyses afforded informative characterizations of the two observed species. An anaerobic sample was prepared containing 150 μM Fe^III^ Lys70Tyr cyt P460 and 700 μM HNO at pH 8.0 and 25 °C. The sample was incubated for 3 min and frozen in liquid N_2_. The EPR spectrum of this sample exhibited *g*-values of 2.09, 2.02, and 1.98 with corresponding ^14^N hyperfine values of 45, 47, and 40 MHz, respectively. These parameters are characteristic of a heme 6c {FeNO}^7^ species. A second sample was prepared by treating 150 μM Fe^II^ Lys70Tyr cyt P460 with 600 μM NO at pH 8.0 and 25 °C with immediate freezing in liquid N_2_. The EPR spectrum of this sample exhibited *g*-values of 2.09, 2.03, and 2.01 with corresponding ^14^N hyperfine values of 47, 41, and 49 MHz, respectively (Fig. 10). As with the WT experiments, the differences in the two EPR spectra are consistent with a conversion from a 6c to a 5c {FeNO}^7^. The Lys70Tyr also does not exhibit a 9-line super hyperfine splitting in the 6c {FeNO}^7^. The correlated EPR and UV-vis absorption spectra indicate that the reaction of Lys70Tyr Fe^III^ cyt P460 with HNO forms a 6c {FeNO}^7^, which decays slowly to a 5c {FeNO}^7^.

Data for the Fe K-edge absorption near-edge regions and EXAFS region of the mutant 5c {FeNO}^7^ species were collected for comparison with that of the WT. The Lys70Tyr mutant also exhibited a pre-edge feature near 7113 eV with intensity similar to that seen in the WT 5c {FeNO}^7^ spectrum. This result is consistent with decreased centrosymmetry at the Fe center resulting from the decrease in coordination number from the dissociation of the axial His (Fig. S6†). EXAFS data were collected to determine the bond distances of Lys70Tyr cyt P460 5c {FeNO}^7^, and fits of the EXAFS region from a *k* of 2–14 Å^–1^ yielded a Fe–NO bond length of 1.80 Å. As with that of the WT 5c {FeNO}^7^, the EXAFS data fit best with the addition of the axial His as a separate parameter, yielding a bond length of 2.48 Å, which is outside the range of coordination for an Fe–N(His) bond (Fig. S10† and Table 1).

### Rapid, NO dependent His140 dissociation when cross-link removed

**Fig. 11:**
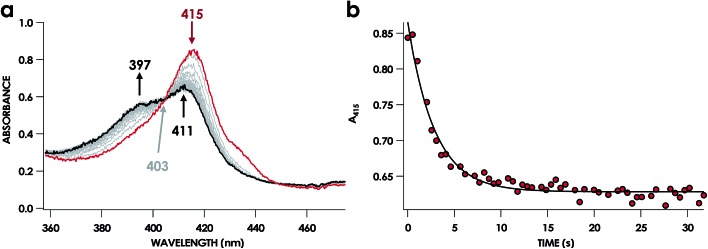
The 32 s stopped-flow UV-vis absorption full-spectral (a) and 415 nm single-wavelength (b) time courses of the reaction of 10 μM Fe^II^ cyt P460 with 100 μM NO in 200 mM HEPES buffer (pH 8.0). In (a), the solid red trace is the spectrum collected immediately after mixing. The black trace is the final spectrum collected at 32 s. Grey spectra were collected in 0.5 s increments. Absorption maxima in nanometers are labeled with colors corresponding to each species. An isosbestic point is labeled in gray. In (c), the black trace is a single exponential (*A*_415_ = *y*_0_ + *A* × e^–*k*_obs_×*t*^) fit to the data, yielding *k*_obs_ = 0.40 s^–1^.

A 6c {FeNO}^7^ species was observed when either Fe^III^ WT or Lys70Tyr cyt P460 was allowed to react with HNO. However, this species was not observed during the reaction of the Fe^II^ form of the mutant with NO, suggesting either the 6c {FeNO}^7^ is never formed or it decays too fast for observation. To differentiate between these possibilities, we monitored the reaction of Fe^II^ Lys70Tyr cyt P460 with NO using stopped-flow UV-vis absorption spectroscopy. The spectral time course exhibited accumulation within 20 ms of absorption features attributed to Lys70Tyr cyt P460 6c {FeNO}^7^ (Fig. 11). This spectrum decayed within 5 s to a new spectrum characteristic of the 5c {FeNO}^7^ species. An isosbestic point at 400 nm suggests direct conversion from the 6c to the 5c {FeNO}^7^ species. These results verify that the 6c {FeNO}^7^ species is generated when Fe^II^ Lys70Tyr cyt P460 reacts with NO and its conversion to the 5c {FeNO}^7^ species is rapid. This 6c-to-5c {FeNO}^7^ conversion is orders of magnitude faster in the reaction when excess NO is present, suggesting that NO induces rapid His140 dissociation. Such rapid NO-dependent His dissociation from 6c heme {FeNO}^7^ species has been previously characterized in several NO-sensing heme proteins.

**Fig. 12:**
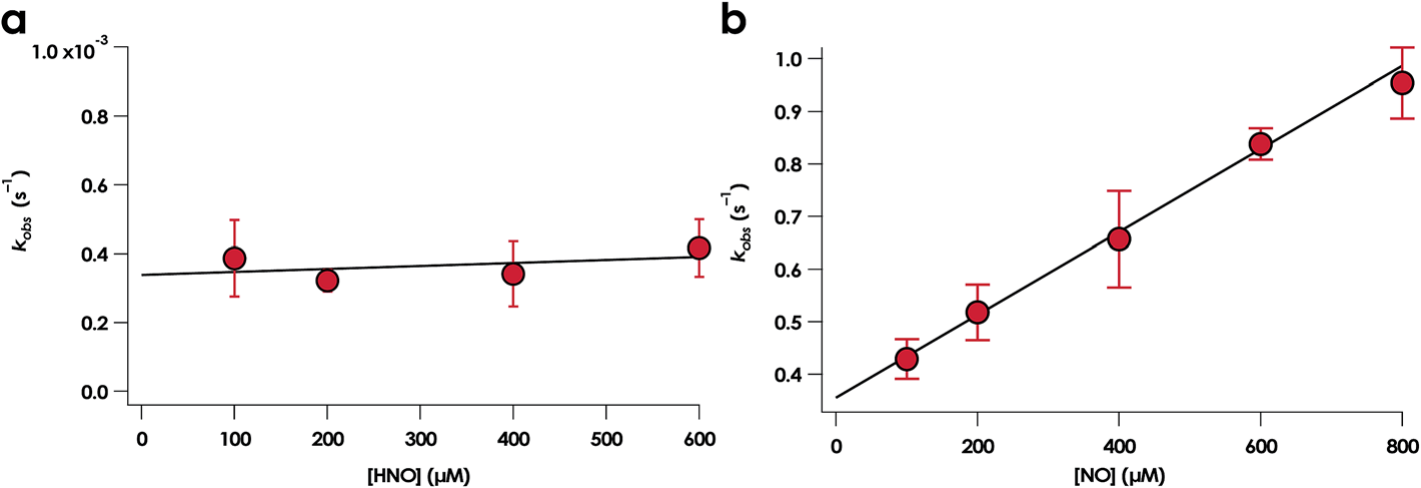
Plots of *k*_obs_ for 6c-to-5c {FeNO}^7^*vs.* HNO concentration (a) and NO concentration (b). The corresponding rate constants are *k*_His-off_ = 3.8 ± 0.9 × 10^–4^ s^–1^ and *k*_His-off(NO)_ = 790 ± 80 × M^–1^ s^–1^ for HNO and NO, respectively.

Rate constants were determined for the NO-independent and NO-dependent His140 dissociation pathways of Lys70Tyr cyt P460 6c {FeNO}^7^. The NO-independent rate constant, *k*_His-off_, was determined from the reactions of 10 μM Fe^III^ Lys70Tyr cyt P460 with HNO at various concentrations in the range of 100–600 μM. The accumulation and decay of 6c {FeNO}^7^ was monitored at 415 nm using UV-vis absorption spectroscopy. Double-exponential functions were fit to the 415 nm traces. The *k*_obs_ for His140 dissociation was zeroth-order with respect to HNO (Fig. 12a). An averaging of the *k*_obs_ values at all HNO concentrations provided a *k*_His-off_ of 3.8 ± 0.9 × 10^–4^ s^–1^, which is an order of magnitude slower than the *k*_His-off_ measured for WT cyt P460.

The rate constant of the NO-dependent His140 dissociation pathway was measured using stopped-flow UV-vis absorption spectroscopy. The NO-dependent rate constant for His140 dissociation, *k*_His-off(NO)_, was determined from the reactions of 10 μM Fe^II^ Lys70Tyr cyt P460 with 100–800 μM NO. These stopped-flow kinetics experiments were monitored at 415 nm. Because the 6c {FeNO}^7^ species formed completely within the stopped-flow mixing dead time, the kinetic traces were monophasic that were well fit by single exponentials. Extracted values of *k*_obs_ were fit to a linear regression with eqn (1) (Fig. 12b):

1 *k*_obs_ = *k*_His-off(NO)_ [NO] + *k*_app_

The best-fit parameters were a *k*_His-off(NO)_ of 790 ± 80 M^–1^ s^–1^ with a *y*-intercept, or *k*_app_, of 0.36 ± 0.04 s^–1^. The first-order dependence of 6c-to-5c {FeNO}^7^ conversion has been used to support a mechanism involving a hypothetical *trans*-dinitrosyl {Fe(NO)_2_}^8^ intermediate in other heme systems.^34^ Our spectral time course is inconsistent with formation of an intermediate during this conversion. Furthermore, it is unclear from our data what *k*_app_ represents. In our hands, the 6c-to-5c {FeNO}^7^ conversion is irreversible, thus the non-zero value for *k*_app_ likely reports the rate constant of an alternative pathway. One possibility is that this *y*-intercept represents the parallel NO-independent His dissociation pathway, *k*_His-off_. However, this value was independently measured to be vastly slower: 3.7 ± 0.4 × 10^–4^ s^–1^. Another possibility is that the range of NO concentrations surveyed was insufficient to observe saturating behavior; such behavior has been noted in other studies of 6c-to-5c heme {FeNO}^7^ conversion.^35^ Our data clearly show that removal of the cross-link introduces an NO-dependent His140 dissociation pathway. However, more detailed mechanistic work will be required to correctly interpret the observed non-zero *y*-intercept of the NO-dependent His dissociation pathway.

The kinetics thus revealed two distinct pathways for His140 dissociation from the Lys70Tyr cyt P460 6c {FeNO}^7^. One pathway is independent of NO with a first-order rate constant similar to that observed for the WT cyt P460 6c {FeNO}^7^ species while the second pathway is absent in the WT cyt P460. The results of these experiments suggest that in the presence of excess NO, the Lys70 cross-link of the P460 cofactor is necessary to inhibit the NO-dependent pathway, thereby allowing the oxidation of 6c {FeNO}^7^ to {FeNO}^6^. These results identify at least one function of the cross-link characteristic of P460 cofactors.

### Activation analysis of His140 dissociation from WT and Lys70Tyr: insight into the role of the cross-link in 6c-to-5c {FeNO}^7^ conversion

Determination of activation enthalpies (Δ*H*^‡^) and entropies (Δ*S*^‡^) for the NO-independent pathways provided insight into how the Lys70 cross-link influences the His140 dissociation rate in the NO-dependent pathway. The results of the Eyring analysis of NO-independent and NO-dependent pathways for WT and Lys70Tyr cyt P460 are shown in Table 2. For both the WT and Lys70Tyr, the NO-independent pathway is dominated by the dissociation of His140. The 1 kcal mol^–1^ difference in Δ*G*^‡^ between the mutant and the WT proteins (ΔΔ*G*^‡^) accords with the 10-fold smaller rate constant for the NO-independent His dissociation of Lys70Tyr cyt P460 compared with that of the WT. The increased barrier to dissociation can largely be attributed to the increased Δ*H*^‡^ of the mutant dissociation reaction. The difference in Δ*H*^‡^ of 11 kcal mol^–1^ between the Lys70Tyr and WT cyt P460 proteins implies an increased Fe–N(His) bond dissociation energy in the former. The relatively small Δ*S*^‡^ of the WT (0.4 ± 0.3 cal mol^–1^ K^–1^) is surprising for a dissociation mechanism, which would assume an overall gain in Δ*S*^‡^ and therefore a larger Δ*S*^‡^. This increase in Δ*S*^‡^ of 27.3 cal mol^–1^ K^–1^ in the Lys70Tyr contributes to lowering the overall Δ*G*^‡^ of the NO-independent pathway to account for a difference of 1 kcal mol^–1^ rather than the approximately 9 kcal mol^–1^ difference if the mutant and WT shared a similar Δ*S*^‡^. Therefore, this difference in Δ*S*^‡^ suggests one functional contribution of the cross-link (*vide infra*).

**Table 2:** His140 dissociation rate constants and activation parameters^*a*^

Temperature (°C)	Wild-type	Lys70Tyr	
	NO-independent (*k*_His-off_)	NO-independent (*k*_His-off_)	NO-dependent (*k*_His-off(NO)_)
15	0.9 ± 0.1 × 10^–3^ s^–1^	6.6 ± 2.3 × 10^–5^ s^–1^	210 ± 20 M^–1^ s^–1^
20	1.86 ± 0.03 × 10^–3^ s^–1^	2.2 ± 0.1 × 10^–4^ s^–1^	510 ± 40 M^–1^ s^–1^
25	2.9 ± 0.2 × 10^–3^ s^–1^	3.8 ± 0.9 × 10^–4^ s^–1^	790 ± 80 M^–1^ s^–1^
30	6.26 ± 0.14 × 10^–3^ s^–1^	1.23 ± 0.08 × 10^–3^ s^–1^	3190 ± 260 M^–1^ s^–1^
35	1.12 ± 0.05 × 10^–2^ s^–1^	2.80 ± 0.03 × 10^–3^ s^–1^	6550 ± 270 M^–1^ s^–1^
Δ*H*^‡^ (kcal mol^–1^)	20.9 ± 0.3	30.2 ± 2.8	30.5 ± 0.7
Δ*S*^‡^ (cal mol^–1^ K^–1^)	0.4 ± 0.3	27.6 ± 2.2	57.8 ± 2.4
Δ*G*^‡^ (25 °C) (kcal mol^–1^)	20.8 ± 0.1	21.9 ± 0.7	13.2 ± 0.7

^*a*^Errors in rate constants represent the standard deviation of three trials per reaction at each temperature. Eyring plots were weighted in Igor. A propagation of error analysis from the weighted errors of the linear regression fit in Igor was used to calculate the errors in the activation parameters.^37^

The activation parameters were also obtained for the NO-dependent His140 dissociation in Lys70Tyr cyt P460. Both the NO-dependent and NO-independent pathways for Lys70Tyr cyt P460 yield similar Δ*H*^‡^ values. This is consistent with the rate of the 6c-to-5c {FeNO}^7^ conversion for both pathways being dominated by the Fe–His bond dissociation energy. The entropic terms from the NO-dependent and NO-independent pathways differ by approximately 30 cal mol^–1^ K^–1^, a value that commonly attends a unit change in reaction order.^36^

## Conclusions

**Fig. 13:**
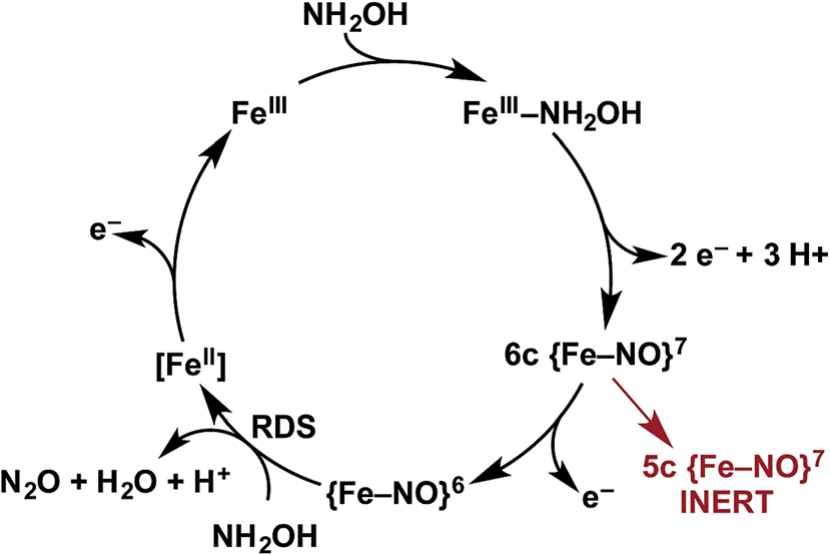
Revised mechanism of NH_2_OH oxidation and formation of N_2_O by cyt P460.

We have identified a 6c {FeNO}^7^ intermediate on the cyt P460 NH_2_OH oxidation pathway (Fig. 13). This species results from an apparent 2-electron oxidation of Fe^III^–NH_2_OH cyt P460. We suspect that Fe^III^–NH_2_OH conversion to 6c {FeNO}^7^ occurs *via* two subsequent and rapid 1-electron oxidation steps with reduction potential inversion between these steps.^38^ Possible 1-electron oxidized intermediates are either the Fe^III^–˙NH_2_OH, invoked as an intermediate for cyt P450 nitric oxide reductase catalysis, or a Fe^II^–HNO species.^39^,^40^ Future studies will pursue the trapping and characterization of these intermediates.

The oxidation of 6c {FeNO}^7^ produces the {FeNO}^6^ intermediate competent for electrophilic attack by NH_2_OH, ultimately resulting in N_2_O release. In the absence of oxidant, the 6c {FeNO}^7^ decays to the inert, off-pathway 5c {FeNO}^7^. However, with oxidant present, the oxidation of 6c {FeNO}^7^ to {FeNO}^6^ proceeds far more swiftly than His dissociation to form the 5c {FeNO}^7^ species. Thus, in the presence of oxidant, catalysis outpaces the formation of the irreversible, off-pathway 5c {FeNO}^7^, thereby preserving active enzyme. From this result, we speculate that the *in vivo* lifetime of cyt P460 is partially dependent on steady-state oxidant concentrations, which should fluctuate with periplasmic O_2_ concentrations. This mechanism could affect active cyt P460 concentrations when AOB transition from oxic to anoxic environments. The bulk enzyme could tolerate short periods of low O_2_ concentration given that 6c-to-5c {FeNO}^7^ conversion requires several minutes. Determining if HAO inactivates in a similar fashion upon oxidant depletion could determine whether our hypothesis also applies to energy-producing reactions in AOB. Indeed, a 5c {FeNO}^7^ species of the HAO P460 cofactor has been observed when the reduced enzyme is treated with NH_2_OH.^17^ However, it remains unclear whether this species is similarly unreactive, as observed for cyt P460 5c {FeNO}^7^ intermediate.

Experiments with the Lys70Tyr cyt P460 showed how the Lys–heme cross-link, the defining characteristic of cyt P460s, is critical for avoiding the off-pathway 5c {FeNO}^7^. This cross-link lengthens the lifetime of the 6c {FeNO}^7^ intermediate (*k*_His-off_ = 2.9 × 10^–3^ s^–1^) compared to that of the other heme proteins. As noted above, the lifetime of the 6c {FeNO}^7^ is related to the *trans* influence exerted by the NO, which weakens the Fe–His140 bond. However, we noted that the Δ*H*^‡^ for His dissociation is larger for the mutant than the WT, implying the mutant has a stronger Fe–N(His) bond strength. The stronger Fe–N(His) bond strength in Lys70Tyr is consistent with the loss of heme ruffling after the removal of the cross-link; the restoration of planarity allows electron delocalization from Fe into the porphyrin π system.^41^ Consequently, Fe becomes less electron-rich, strengthening the Fe–N(His) interaction while weakening the Fe–NO interaction. The elongated Fe–NO distance of the Lys70Tyr 5c {FeNO}^7^ species relative to that of the WT (1.81 Å *vs.* 1.74 Å, respectively) obtained from the EXAFS data could be interpreted as a consequence of the increased Fe–N(His) strength which weakens the σ-bonding donation of the NO to the Fe center thereby lengthening the Fe–N(O) bond.

The 6c {FeNO}^7^ species of the Lys70Tyr variant exhibited a rapid His dissociation with a first-order dependence on NO [*k*_His-off(NO)_ = 790 M^–1^ s^–1^]. The combined data imply that the Lys–heme cross-link renders cyt P460 6c {FeNO}^7^ insensitive to NO. We have proposed that NO_2_^–^ produced during aerobic NH_2_OH oxidation turnover by cyt P460 is a non-enzymatic product; NO dissociates from the {FeNO}^6^ intermediate of the catalytic cycle and reacts with O_2_ to generate the observed NO_2_^–^. In other words, one product of cyt P460 is NO. In the absence of an NO sink, cyt P460 could be surrounded by a local pocket of high NO concentration generated by its own turnover; thus, cyt P460 generates a potential poison to its own catalytic cycle. Furthermore, recent work from our laboratory has established NO as an obligate intermediate in the NH_3_-oxidizing pathway through NH_2_OH oxidation by HAO.^3^ Thus, intracellular NO likely accumulates during oxic metabolism. The cross-link, therefore, appears to be necessary for avoiding cyt P460 catalysis inactivation under conditions with available NO.

NO-dependent 6c-to-5c {FeNO}^7^ conversion is also characteristic of the human NO-sensing protein sGC. Along with its bacterial counterparts, these NO sensors are collectively known as H-NOX (Heme NO/O_2_ binding) proteins because they contain an H-NOX domain, a b-heme with an axially bound His. Downstream signaling is conferred through a partner protein, or in sGC, an attached guanylate cyclase domain. NO binds to the Fe^II^–heme to form a 6c {FeNO}^7^ species that rapidly converts to a 5c {FeNO}^7^ species. The ensuing His dissociation induces a protein conformational change that activates the cyclase domain, thereby initiating the signaling cascade. Three hypotheses have been offered to explain the observed NO dependence on the rate of the 6c-to-5c {FeNO}^7^ conversion: (1) NO binding at an allosteric site promotes His dissociation, (2) a second NO molecule replaces the axial His to form a *trans*-dinitrosyl intermediate, or (3) nucleophilic attack on the {FeNO}^7^ intermediate by a second NO molecule results in N–N bond formation, which in turn results in His dissociation.^18^ However, NO dependence on His dissociation has also been observed for our cyt P460 mutant, cyt c′-α and an engineered heme/non-heme nitric oxide reductase, none of which are related to H-NOX proteins.^42^,^43^ These non-H-NOX proteins are unlikely to contain the same allosteric site, therefore, we can rule out hypothesis 1 as a general mechanism. Biological N–N bond formation is preceded by diferrous-dinitrosyl ([{FeNO}^7^]_2_), Fe^III^–˙NH_2_OH, or {FeNO}^6^ intermediates.^13^,^40^,^42^,^44^–^46^ Although we cannot rule out hypothesis 3, N–N bond formation *via* the nucleophilic attack of NO on an {FeNO}^7^ species has not yet been observed in a biological system. In support of hypothesis 2, the crystal structure of a cyt c′-α protein from *Alcaligenes xylosoxidans*, which also exhibits NO-dependent 6c-to-5c {FeNO}^7^ conversion, shows that NO is bound to the proximal side of the heme.^47^ Furthermore, kinetic evidence exists for an intermediate species between the 6c and 5c {FeNO}^7^ of a *Nostoc* sp. H-NOX protein.^48^ This intermediate was proposed to be *trans*-dinitrosyl. We saw no evidence for an intermediate in our experiments, which suggests that the mechanism for Lys70Tyr cyt P460 differs from those proposed for H-NOX. Therefore, to elucidate this NO-dependent mechanism, we will need to perform further kinetic and intermediate trapping studies. For the purpose of the current study, the relevant result is that the removal of the cross-link causes the rate of His dissociation to be dominated by a NO-dependent pathway that is absent in the native cyt P460.

The lack of a NO-dependent pathway for WT cyt P460 could be due to decreased accessibility at the proximal side of the heme, precluding the binding of the second NO molecule. For the NO-independent His140 dissociation pathway, the WT cyt P460 has a Δ*S*^‡^ term (0.4 ± 0.3 cal mol^–1^ K^–1^) that is smaller than that for Lys70Tyr cyt P460 (27.6 ± 2.2 cal mol^–1^ K^–1^). A similarly large Δ*S*^‡^ was found for His dissociation from *A. xylosoxidans* c′ {FeNO}^7^.^49^ We propose that the change in the Δ*S*^‡^ term for the WT cyt P460 reflects the degrees of freedom of the His140 ligand. By this hypothesis, the discrepancy between the Δ*S*^‡^ terms may indicate that the His140 pocket in WT cyt P460 is more rigid, which could decrease NO accessibility on the proximal side of the heme and preclude the NO-dependent His dissociation pathway. Thus, the cyt P460 cross-link “locks” the axial His of the 6c {FeNO}^7^ species, thereby slowing its dissociation rate. This impediment allows the oxidation of 6c {FeNO}^7^ to {FeNO}^6^ to kinetically outcompete protein inactivation.

Our results suggest that the Lys70Tyr cyt P460 mutant can be used as a model for signal transduction by H-NOX proteins. The increased Δ*S*^‡^ term in the cyt P460 mutant may reflect a less rigid pocket surrounding the axial His. In contrast, H-NOX proteins rely on protein conformational changes induced by the formation of the 5c {FeNO}^7^ complex to activate signal transduction. Locking the protein conformation would be detrimental to the activation of cyclase activity or interactions with partner signaling proteins. Therefore, the increased degrees of motion in the His pocket may be necessary for signal transduction. To test this hypothesis, mutagenesis studies that perturb the Fe–His interaction will be carried out to explore the effects of His140 pocket alterations on the dissociation of the axial His on other cross-link deficient cyt P460 {FeNO}^7^ mutants.

Our results also show that the 6c {FeNO}^7^ species can be independently generated by treating either Fe^III^ with HNO or Fe^II^ with NO. For the Lys70Tyr cyt P460, these treatments result in either a slow, HNO-concentration-independent His dissociation or a rapid, NO-concentration-dependent His dissociation, respectively. HNO and NO are known to exhibit orthologous physiological effects in humans.^50^ The observed differences in the rate laws for the dissociation of axial His from 6c {FeNO}^7^ in the presence of HNO or NO may provide insight into the divergent signaling pathways and orthologous physiological effects.

In summary, we have characterized a 6c {FeNO}^7^ species on the NH_2_OH oxidation pathway of cyt P460. This species can undergo axial His dissociation to yield an off-pathway 5c {FeNO}^7^ species. Kinetic analysis of WT and Lys70Tyr cyt P460 proteins show that the Lys–heme cross-link of the WT protein eliminates a NO-dependent pathway toward 6c-to-5c {FeNO}^7^ conversion. Avoidance of this pathway appears to be critical for preserving cyt P460 activity in the periplasmic space of AOB, which necessarily includes NO as an obligate nitrification intermediate. Eyring analyses of the 6c-to-5c {FeNO}^7^ conversion were compared to gain insight into how the Lys–heme cross-link increases this activation barrier. Compared with the WT protein pathways, the NO-independent pathway of the cross-link deficient mutant has a higher activation entropy. We interpret this observation as evidence that the Lys–heme cross-link confers rigidity to the pocket surrounding the axial His in the WT and propose that this rigidity decreases the number of possible His dissociation pathways. We contend that in addition to obviation of NO-dependent His dissociation, another role for the Lys–heme cross-link is protective: it disfavors 6c-to-5c {FeNO}^7^ conversion and consequent inactivation of cyt P460.

## Conflicts of interest

There are no conflicts to declare.

## Supplementary Material

Supplementary information
