# Supplementary material for: Influences of the heme-lysine crosslink in cytochrome P460 over redox catalysis and nitric oxide sensitivity
Source: Chem Sci. 2017 Nov 7;9(2):368–79. doi: 10.1039/c7sc03450d (PMC5872139; doi:10.1039/c7sc03450d)
Supplement: Supplementary file 1 [file SC-009-C7SC03450D-s001.pdf]

Electronic Supplementary Material (ESI) for Chemical Science.
This journal is © The Royal Society of Chemistry 2017

The Lysine Cross-Link to Heme P460 Obviates NO-Dependent Histidine-Dissociation from *Nitrosomonas europaea* Cytochrome P460 $\{FeNO\}^7$

SUPPORTING INFORMATION

*Avery C. Vilbert, Jonathan D. Caranto, and Kyle M. Lancaster**

Department of Chemistry and Chemical Biology, Baker Laboratory, Cornell University, Ithaca, NY 14853

TABLE OF CONTENTS

Supplementary Figures S2

Supplementary Tables S10

Supplementary Figures

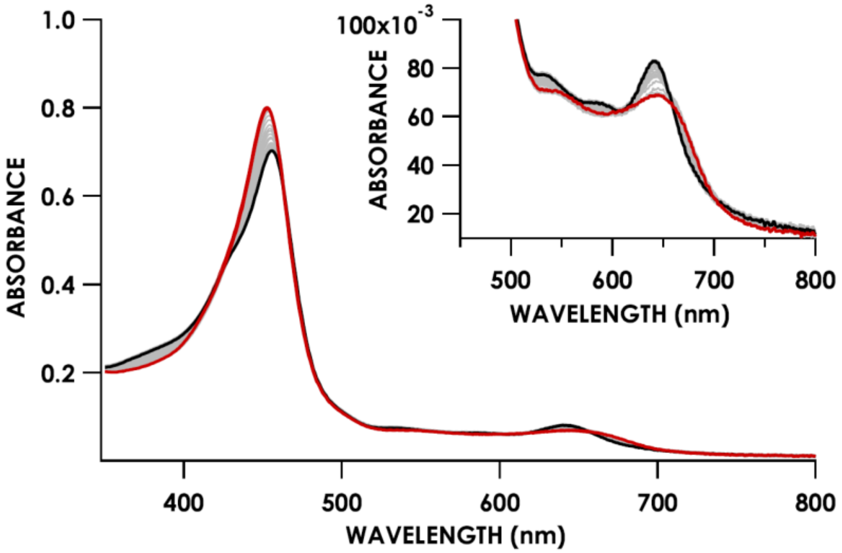

Figure S1. UV-vis absorption full-spectral scans of the reaction of 15µM 6c $\{FeNO\}^7$ and 10mM NH_2OH in 200mM HEPES pH 8.0. The solid red trace corresponds to the 6c $\{FeNO\}^7$ after addition of 10mM NH_2OH. Grey spectra are collected in 30s increments for 30 min and the black spectra is the final species corresponding to the 5c $\{FeNO\}^7$. Inset highlights the q band region after the addition of NH_2OH and corresponds to the transition from the 6c to 5c $\{FeNO\}^7$..

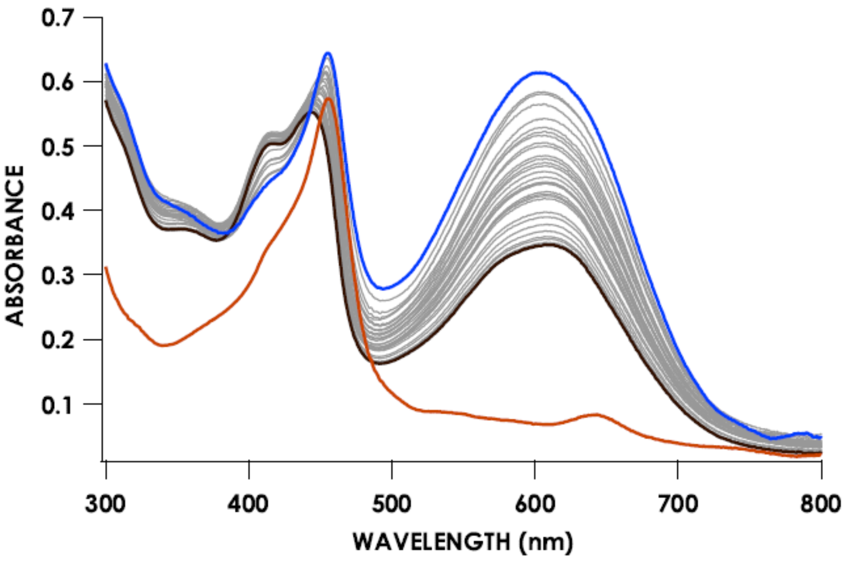

Figure S2. UV-vis absorption full-spectral scans of the reaction of 5c $\{FeNO\}^7$ species formed by treatment of 15µM Fe^{III} and 100µM of the HNO donor $Na_2N_2O_3$ and allowed to react for 30 mins in 200mM HEPES pH 8.0. The solid red trace corresponds to the 5c $\{FeNO\}^7$ prior to the addition of the oxidant dichloroindolphenol (DCPIP). The solid blue trace is immediately after addition of 1mM DCPIP. Grey spectra are collected in 30s increments for 20 min and the black spectra is the final species with a split soret max at 413 nm and 442 nm.

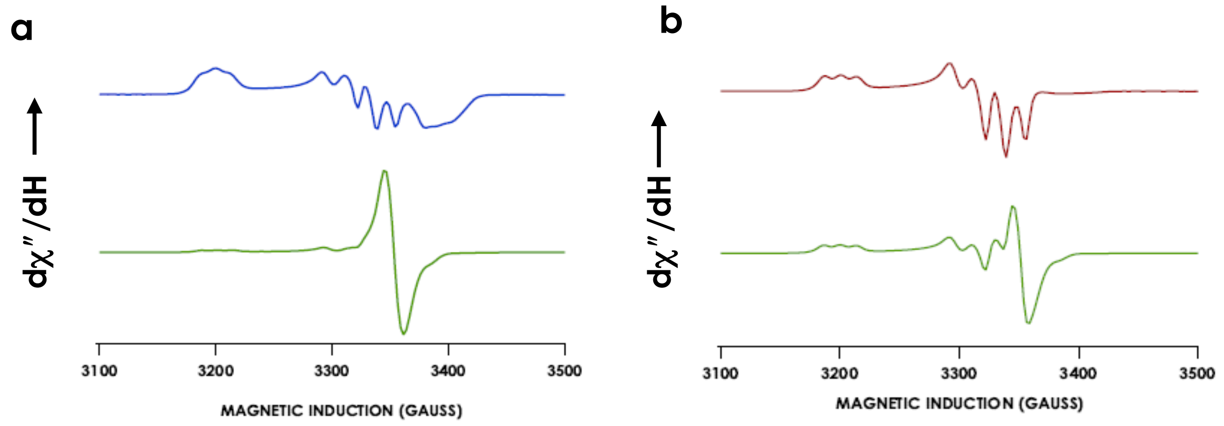

Figure S3. EPR spectra of 200 µM P460 Fe^{III} cyt P460 with the addition of 1mM of the HNO donor $Na_2N_2O_3$ at room temperature for a) 2 min (blue trace) or b) 30 min (red trace) and subsequently reacted with 800 µM of the oxidant phenazine methosulfate (green traces).

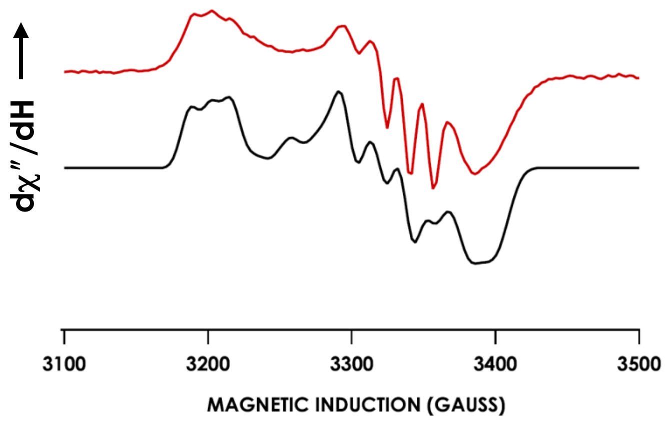

Figure S4. EPR spectrum shown in red of 200 µM P460 treated with 1 mM NH_2OH and 1 mM dicholorophenolindophenol (DCPIP) in 200 mM HEPES buffer (pH 8.0). The sample was frozen 2 min after the consumption of oxidant. The black spectrum is the corresponding simulation of the Fe^{III}-NH_2OH oxidized species and is consistent with the 6c $\{FeNO\}^7$ generated from either addition of HNO or NO to Fe^{III} and Fe^{II}, respectfully.

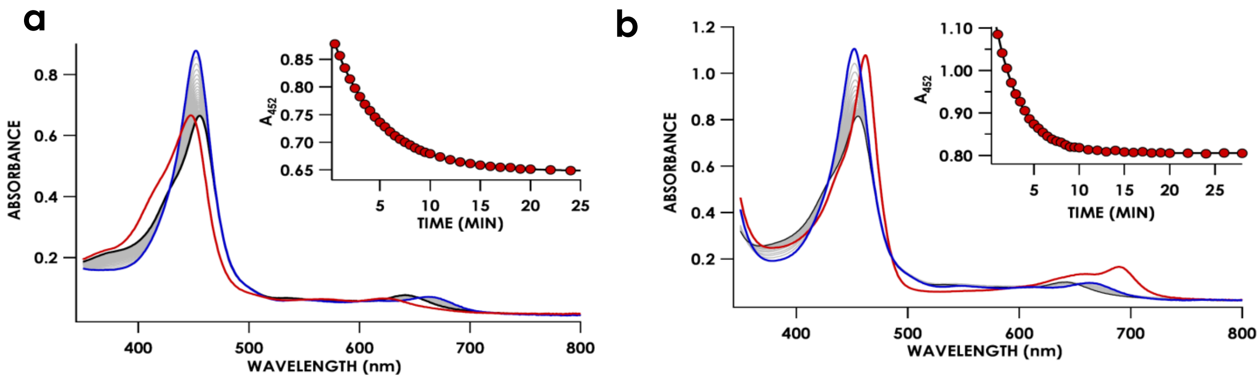

Figure S5. UV-vis absorption full-spectral scans of the reaction of cyt P460 15µM (a) Fe^{III} and 100µM of the HNO donor $Na_2N_2O_3$ or (b) Fe^{II} with 100µM NO (50µM Proli-NONOate). The red trace is the initial species of (a) Fe^{III} or (b) Fe^{II} .The blue trace is after the addition of (a) HNO or (b) NO and black is the final trace of the 5c $\{FeNO\}^7$. The insert is the corresponding single wavelength time course following the absorbance at 452 nm.

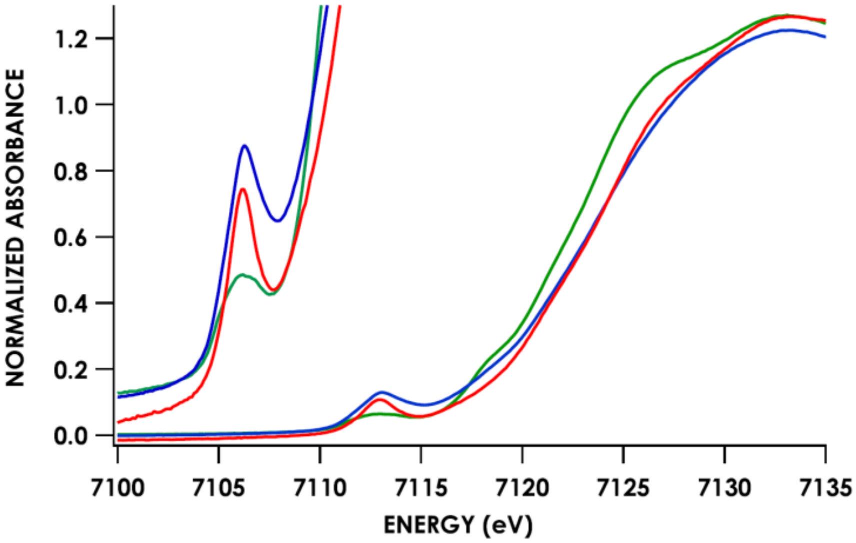

Figure S6. Fe-K edge X-ray absorption spectra (XAS) obtained at 10 K of 1mM Fe^{II} Lys70Tyr treated with 10mM NO immediately frozen to form the Lys70Tyr 5c $\{FeNO\}^7$ species (red trace). The Lys70Tyr 5c $\{FeNO\}^7$ species is compared to the 6c (green trace) and 5c (blue trace) WT cyt P460 $\{FeNO\}^7$ species. All samples were glassed in 200mM HEPES buffer pH 8.0 with 25% v/v glycerol. The 1s → 3d pre-edge feature is at 7113.3 eV.

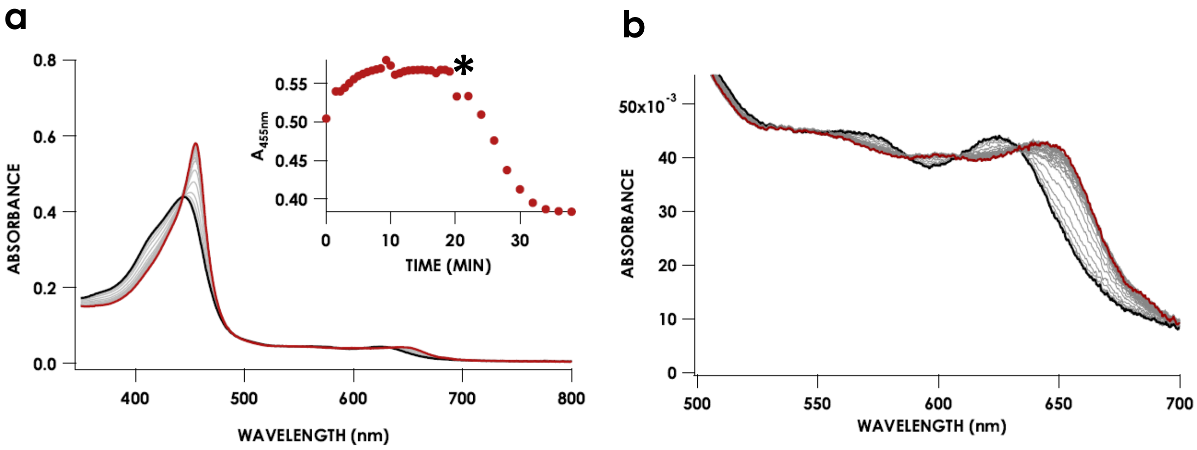

Figure S7. UV-vis absorption full-spectral scans (a) of the reaction of cyt P460 15μM Fe^{III} and 100μM of the HNO donor $Na_2N_2O_3$ after the addition of 100 μM $Ru(NH_3)_6Cl_3$ (red trace) to form the $\{FeNO\}^6$ intermediate. The gray spectra were collected in 30s increments for 40 min and the black spectra corresponds to the Fe^{III} complex. The insert is the corresponding single wavelength time course following the absorbance at 455 nm and the * indicates the addition of 1mM HA after maximal formation of the $\{FeNO\}^6$ species. (b) Highlights the q bands following the transition from the $\{FeNO\}^6$ to the Fe^{III} complex.

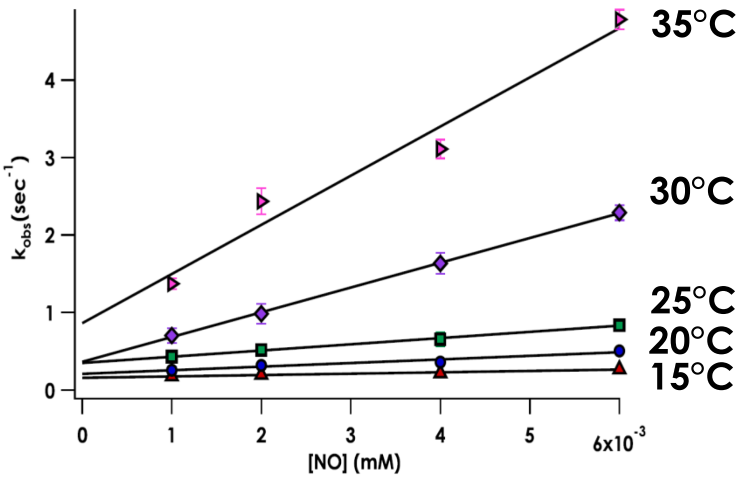

Figure S8. Plots of k_{obs} versus NO concentration as a function of temperature for the reaction of Fe^{II} Lys70Tyr cyt P460 and NO.

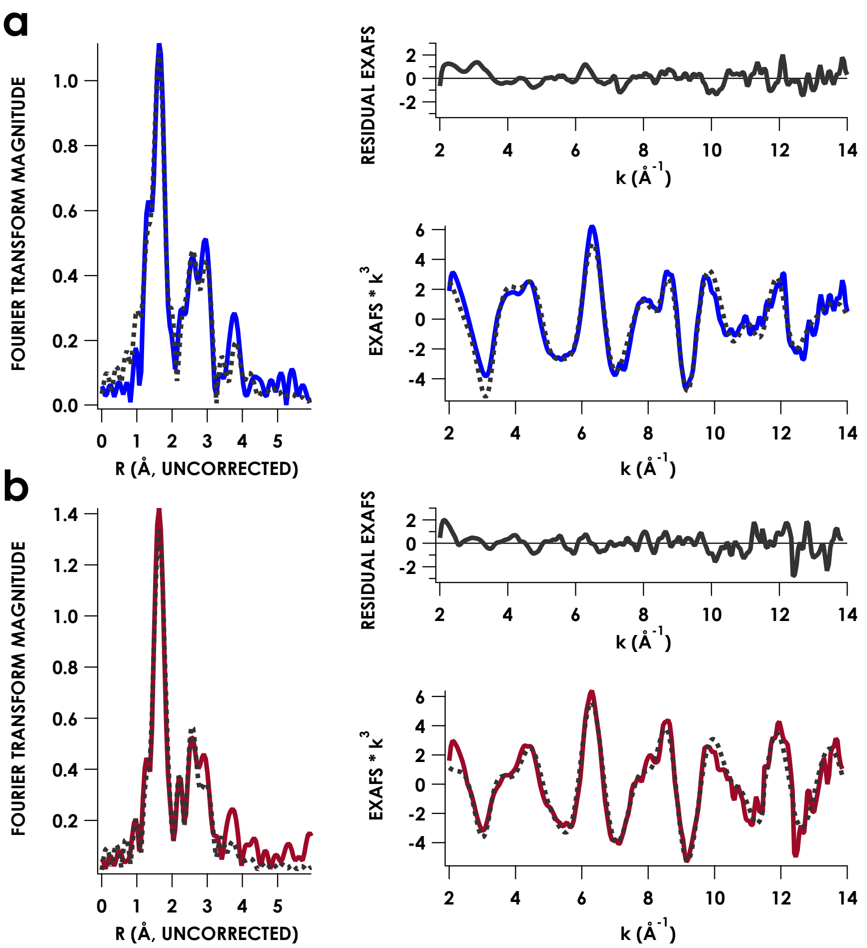

Figure S9. Fe K-edge EXAFS data obtained at 10 K for the 6c (a) and 5c (b) cyt P460 $\{FeNO\}^7$ intermediates in glassed 200 mM HEPES buffer (pH 8.0) containing 25% v/v glycerol. Experimental data are plotted as solid lines; fits are dotted lines.

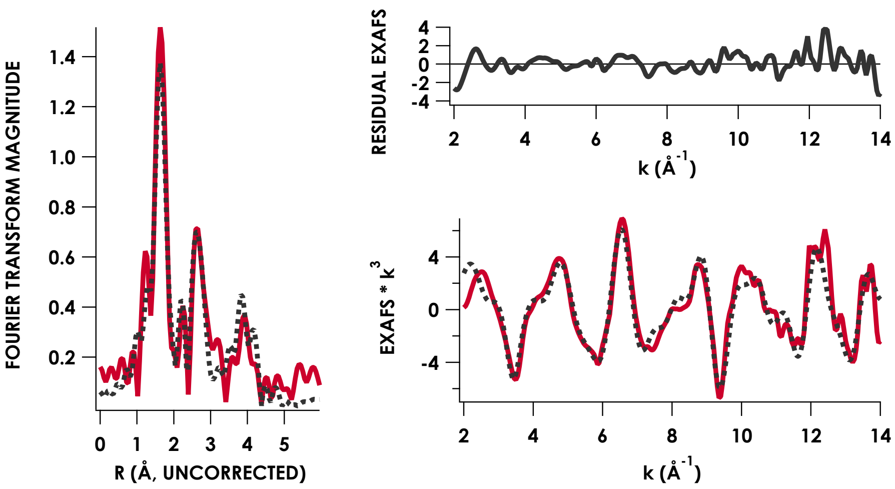

Figure S10. Fe K-edge EXAFS data obtained at 10 K for Lys70Tyr cyt P460 5c $\{FeNO\}^7$ in glassed 200 mM HEPES buffer (pH 8.0) containing 25% v/v glycerol. Experimental data are plotted as solid lines; fits are dotted lines.

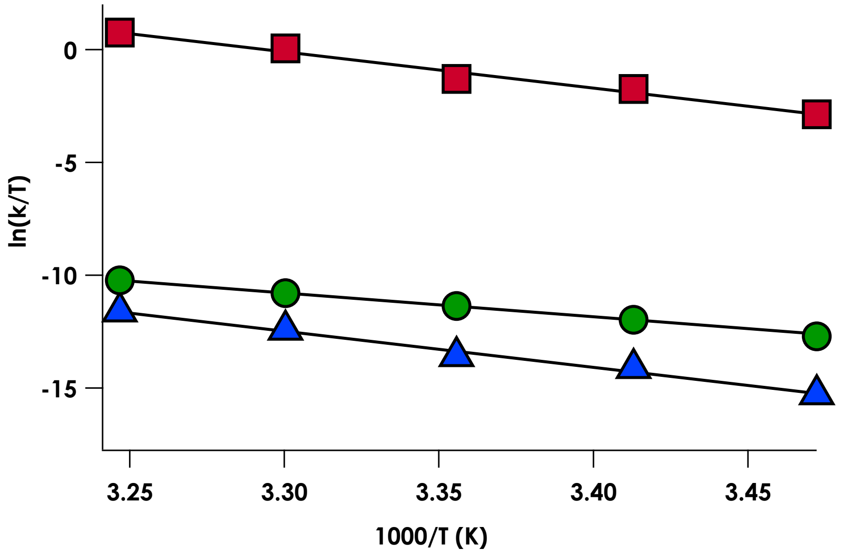

Figure S11. Eyring plots of ln($k_{His\text{-}off}$/T) for WT (green circles) and Lys70Tyr (blue triangles) cyt P460 $\{FeNO\}^7$ species and ln[$k_{His\text{-}off(NO)}$/T] for Lys70Tyr cyt P460 (red squares) vs 1000/T. Rate constants and activation parameters appear in Table 3.

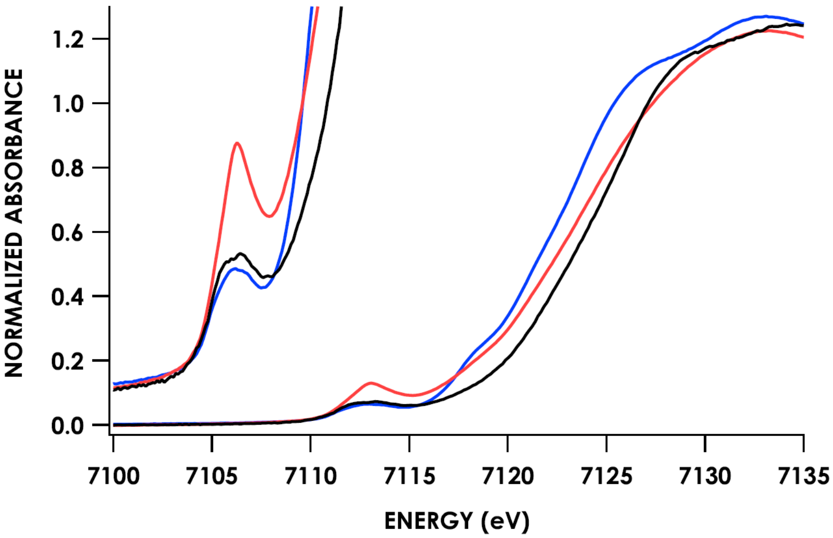

Figure S12. Fe-K edge X-ray absorption spectra (XAS) obtained at 10 K of 1mM Fe^{III} WT cyt P460 treated with 10mM NO immediately frozen to form the $\{FeNO\}^6$ species (black trace). The $\{FeNO\}^6$ species is compared to the 6c (blue trace) and 5c (red trace) WT cyt P460 $\{FeNO\}^7$ species. All samples were glassed in 200mM HEPES buffer pH 8.0 with 25% v/v glycerol. The 1s → 3d pre-edge feature is at 7113.3 eV.

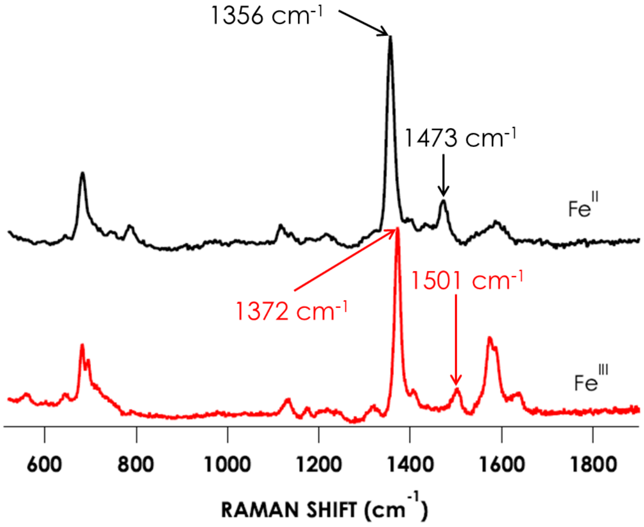

Figure **S13**. The rR spectra obtained via near-resonance excitation with Soret absorption band: λ_{ex} = 405.0 nm (20 mW) for Ly70Tyr Fe^{II} (black) and Fe^{III} (red) the ν(4) and ν(3) are labeled above.

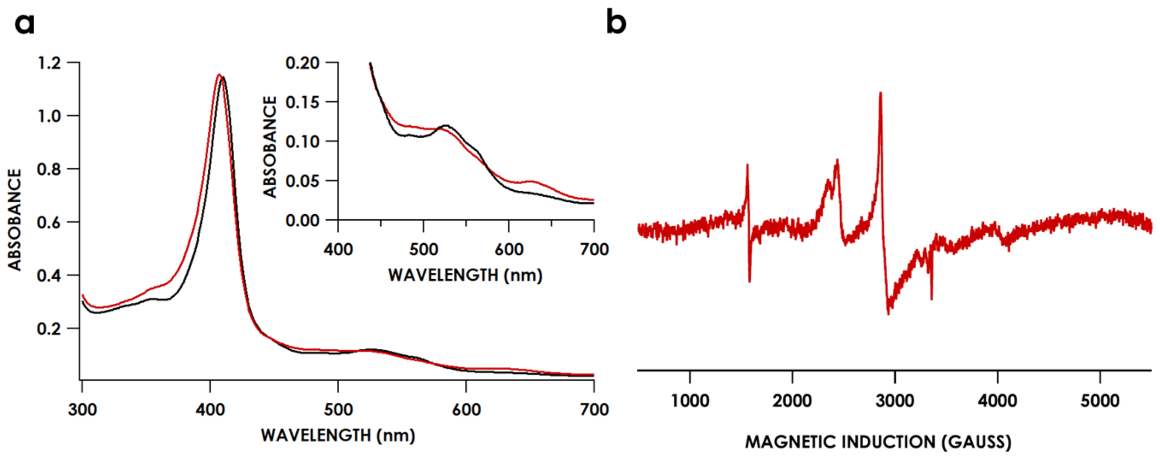

Figure S14. UV-vis absorption spectrum (a) of 10μM K70Y with the addition of 20mM NH_2OH in 200mM HEPES buffer pH 8.0 and EPR spectrum (b) of 200 μM Lys70Tyr treated with 100 mM NH_2OH prepared in 200mM HEPES buffer pH 8.0 with 25% glycerol.

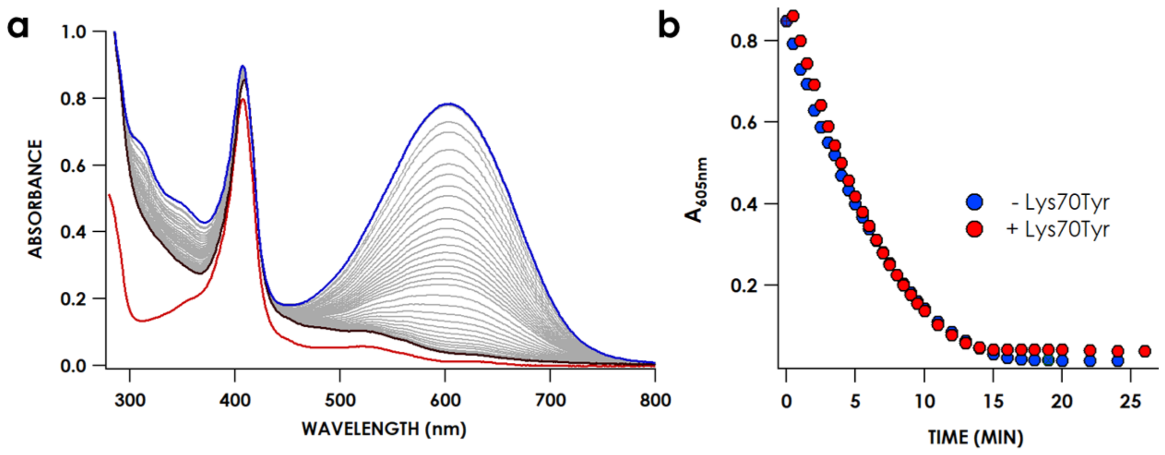

Figure S15. UV-vis absorption spectrum (a) of 10μM K70Y with 15mM NH_2OH and the addition of 100μM DCPIP in 200mM HEPES buffer pH 8.0 and (b) the corresponding single wavelength time course following the consumption of DCPIP with conditions of 100 μM DCPIP and 15mM NH_2OH either in the presence or absence of 10μM K70Y.

Supplementary Tables

Table S1. EXAFS simulations for cyt P460 6c $\{FeNO\}^7$ EXAFS data were fit with EXAFSPAK using paths calculated by FEFF7. Distance and Debye-Waller factors were allowed to float while, coordination numbers were held constant. Goodness of fit is determined by F, defined as $[(EXAFS_{abs} - EXAFS_{calc})_i)^2/n]^{1/2}$. Fe-$N_p$ denotes the pyrrole nitrogens of the porphyrin, Fe-C_α denotes the α carbons of the porphyrin, Fe-C_{meso} denotes the meso carbons of the porphyrin and Fe-C_β denotes the β carbons of the porphyrin.

Fit	Path	Coordination #	R(Å)	±	σ^2	±	F
1	Fe-N_p	5	2.038	0.0047	0.0062	0.0004	60.40%
2	Fe-N_p	5	2.029	0.0056	0.0049	0.0004	
	Fe-N(NO)	1	1.849	0.0185	0.0054	0.0021	58.99%
3	Fe-N_p	5	2.043	0.0028	0.0060	0.0003	
	Fe-N(NO)	1	1.647	0.0307	0.0252	0.0070	
	Fe-C_α	8	3.041	0.0051	0.0053	0.0005	43.02%
4	Fe-N_p	5	2.044	0.0023	0.0061	0.0002	
	Fe-NO	1	1.650	0.0270	0.0266	0.0059	
	Fe-C_α	8	3.042	0.0045	0.0058	0.0004	
	Fe-C_{meso}	4	3.376	0.0045	0.0058	0.0004	33.05%
5	Fe-N_p	5	2.037	0.0027	0.0048	0.0003	
	Fe-C_α	8	3.020	0.0045	0.0063	0.0005	
	Fe-C_{meso}	4	3.364	0.0044	0.0015	0.0004	
	Fe-N(NO)	1	1.862	0.0108	0.0058	0.0013	
	Fe-C_β	8	4.446	0.0122	0.0066	0.0013	32.45%
6	Fe-N_p	5	2.037	0.0024	0.0050	0.0002	
	Fe-C_α	8	3.026	0.0041	0.0063	0.0004	
	Fe-C_{meso}	4	3.364	0.0041	0.0016	0.0004	
	Fe-N(NO)	1	1.858	0.0117	0.0074	0.0015	
	Fe-C_β	16	4.334	0.0084	0.0006	0.0009	29.58%

Table S2. EXAFS simulations for cyt P460 5c $\{FeNO\}^7$ EXAFS data were fit with EXAFSPAK using paths calculated by FEFF7. Distance and Debye-Waller factors were allowed to float while, coordination numbers were held constant. Goodness of fit is determined by F, defined as $[(EXAFS_{abs} - EXAFS_{calc})_i)^2/n]^{1/2}$. Fe-$N_p$ denotes the pyrrole nitrogens of the porphyrin, Fe-Cα denotes the α carbons of the porphyrin, Fe-C_{meso} denotes the meso carbons of the porphyrin and Fe-C_β denotes the β carbons of the porphyrin.

Fit	Path	Coordination #	R(Å)	±	σ^2	±	F
1	Fe-N_p	4	2.020	0.0034	0.0028	0.0002	55.04%
2	Fe-N_p	4	2.023	0.0022	0.0028	0.0002	
	Fe-C_α	8	3.036	0.0052	0.0050	0.0005	41.28%
3	Fe-N_p	5	2.023	0.0025	0.0040	0.0002	
	Fe-C_α	8	3.033	0.0056	0.0050	0.0006	45.91%
4	Fe-N_p	4	2.022	0.0022	0.0027	0.0005	
	Fe-C_α	8	3.036	0.0050	0.0051	0.0005	
	Fe-N(NO)	1	1.723	0.0233	0.0161	0.0042	38.05%
5	Fe-N_p	4	2.024	0.0021	0.0028	0.0002	
	Fe-C_α	8	3.039	0.0049	0.0052	0.0004	
	Fe-N(NO)	1	1.715	0.0226	0.0171	0.0039	
	Fe-C_{meso}	4	3.389	0.0151	0.0053	0.0011	33.48%
6	Fe-N_p	4	2.025	0.0020	0.0028	0.0002	
	Fe-C_α	8	3.041	0.0047	0.0053	0.0004	
	Fe-N(NO)	1	1.703	0.0181	0.0181	0.0041	
	Fe-C_{meso}	4	3.395	0.0048	0.0050	0.0011	
	Fe-N(His)	1	2.533	0.0137	0.0042	0.0015	32.42%
8	Fe-N_p	4	2.021	0.0020	0.0027	0.0001	
	Fe-C_α	8	3.032	0.0048	0.0054	0.0004	
	Fe-N(NO)	1	1.742	0.0255	0.0189	0.0045	
	Fe-C_{meso}	4	3.382	0.0087	0.0043	0.0009	
	Fe-C_β	16	4.381	0.0200	0.0059	0.0027	
	Fe-N(His)	1	2.524	0.0125	0.0039	0.0013	31.23%
9	Fe-N_p	4	2.021	0.0020	0.0027	0.0002	
	Fe-C_α	8	3.028	0.0047	0.0053	0.0004	
	Fe-N(NO)	0.75	1.735	0.0227	0.0129	0.0036	
	Fe-C_{meso}	4	3.384	0.0088	0.0044	0.0009	
	Fe-C_β	16	4.385	0.1995	0.0060	0.0028	
	Fe-N(His)	1	2.525	0.0124	0.0038	0.0013	30.25%

Table S3. EXAFS simulations for Lys70Tyr cyt P460 5c $\{FeNO\}^7$ EXAFS data were fit with EXAFSPAK using paths calculated by FEFF7. Distance and Debye-Waller factors were allowed to float while, coordination numbers were held constant. Goodness of fit is determined by F, defined as $[(EXAFS_{abs} - EXAFS_{calc})_i)^2/n]^{1/2}$. Fe-$N_p$ denotes the pyrrole nitrogens of the porphyrin, Fe-C_α denotes the α carbons of the porphyrin, Fe-MS denotes carbon nitrogen multiscatters and Fe-C_β denotes the β carbons of the porphyrin.

Fit	Path	Coordination #	R(Å)	±	σ^2	±	F
1	Fe-N_p	4	1.998	0.0038	0.0033	0.0003	60.54%
2	Fe-N_p	4	2.009	0.0029	0.0035	0.0003	
	Fe-C_α	8	2.977	0.0061	0.0039	0.0005	55.84%
3	Fe-N_p	5	2.009	0.0032	0.0050	0.0035	
	Fe-C_α	8	2.968	0.0066	0.0038	0.0006	61.34%
4	Fe-N_p	4	1.990	0.0038	0.0030	0.0003	
	Fe-C_α	8	3.002	0.0054	0.0039	0.0005	
	Fe-N(NO)	1	1.776	0.0156	0.0056	0.0018	52.44%
5	Fe-N_p	4	1.992	0.0037	0.0030	0.0003	
	Fe-C_α	8	3.000	0.0055	0.0041	0.0006	
	Fe-N(NO)	1	1.788	0.0194	0.0073	0.0025	
	Fe-N(His)	1	2.502	0.0156	0.0023	0.0016	51.01%
6	Fe-N_p	4	1.995	0.0030	0.0030	0.0003	
	Fe-C_α	8	3.000	0.0044	0.0037	0.0004	
	Fe-N(NO)	1	1.807	0.0185	0.0086	0.0026	
	Fe-N(His)	1	2.490	0.0115	0.0019	0.0012	
	Fe-MS	16	3.233	0.0132	0.0025	0.0017	40.66%
7	Fe-Np	4	1.991	0.0025	0.0028	0.0002	
	Fe-C_α	8	2.997	0.0038	0.0038	0.0003	
	Fe-N(NO)	1	1.805	0.0141	0.0069	0.0018	
	Fe-N(His)	8	2.483	0.0117	0.0026	0.0012	
	Fe-MS	16	3.224	0.0114	0.0022	0.0014	
	Fe-C_β	8	4.341	0.0055	0.0020	0.0005	35.66%
